# Adverse childhood circumstances and cognitive function in middle-aged and older Chinese adults: Lower level or faster decline?^☆^

**DOI:** 10.1016/j.ssmph.2021.100767

**Published:** 2021-03-15

**Authors:** Zhuoer Lin, Xi Chen

**Affiliations:** a Department of Health Policy and Management, Yale School of Public Health, New Haven, CT, USA; b Department of Economics, Yale University, New Haven, CT, USA; c Alzheimer's Disease Research Center, New Haven, CT, USA

**Keywords:** Childhood circumstances, Life course factors, Cognitive aging, Education

## Abstract

We examine the long-term relationship between childhood circumstances and cognitive aging. In particular, we differentiate the level of cognitive deficit from the rate of cognitive decline. Applying a linear mixed-effect model to three waves of China Health and Retirement Longitudinal Surveys (CHARLS 2011, 2013, 2015) and matching cognitive outcomes to CHARLS Life History Survey (2014), we find that key domains of childhood circumstances, including family socioeconomic status (SES), neighborhood cohesion, friendship, and health conditions, are significantly associated with both the level of cognitive deficit and the rate of decline. In contrast, childhood neighborhood safety only affects the level of cognitive deficit. Childhood relationship with mother only affects the rate of cognitive decline. The effects of adverse childhood circumstances are generally larger on level of cognitive deficit than on rate of cognitive decline. Moreover, education plays a more important role in mediating the relationships compared to other later-life factors. These findings suggest that exposure to disadvantaged childhood circumstances can exacerbate cognitive deficit as well as cognitive decline over time, which may be partially ameliorated by educational attainment.

## Highlights

•Exposure to disadvantaged childhood circumstances may exacerbate the process of cognitive aging.
• Neighborhood safety affects the level of cognitive deficit, relationship with mother affects the rate of cognitive decline.
• Childhood SES, neighborhood cohesion, friendship, and health conditions can affect both components of cognitive aging.
• Effects of adverse childhood circumstances are mostly larger on level than those on rate.
• Education plays a critical role in mediating the relationship between adverse childhood circumstances and cognitive aging.

## 1 Introduction

The varying trajectories of health and well-being of older adults may result from a complex interaction of social, environmental, and physiological factors over the life course (Chatterji et al., 2015). With the accretion of knowledge in health and aging, there is converging interest in a life course perspective on later life health trajectories from different disciplines (Burton-Jeangros et al., 2015). Cumulative evidence has suggested the lasting impacts of life course circumstances, especially those in early life (Liu et al., 2019). In particular, adverse early-life exposure may not only affect health directly, but influence individuals’ ability to adapt and to exercise self-control, exacerbating vulnerability to health shocks in old age (Burton-Jeangros et al., 2015; Huber et al., 2011).

A sizable body of research has focused on the long-term health impacts of childhood circumstances to inform interventions in earlier stages. They show that during childhood, socioeconomic status (SES) (Katikireddi, 2016; Moody-Ayers et al., 2007), health and nutritional conditions (Almond & Mazumder, 2011; McEniry et al., 2008), community environment (Aneshensel & Sucoff, 1996; Shen, 2014), and other childhood exposures (Black et al., 2016; Simon, 2016) are associated with various aspects of health in later life. Although previous studies have revealed multiple pathways through which childhood circumstances may affect physical health, mental health, and frailty status in later life, direct evidence on the relationship between childhood circumstances and cognitive aging is still partial and limited.

Given the essential roles of cognitive functioning play in later-life, this knowledge gap may impede targeted interventions, thus requiring thorough investigations. In fact, the impacts of childhood circumstances on cognitive aging can be large and profound. Several hypothetical models have implied the persisting influence childhood circumstances may have on cognition across the life course. To begin with, the *critical period model* points out the critical impacts of prenatal, postnatal, and early childhood exposures on brain development and cognitive reserve (Lynch & Smith, 2005). For example, gestational and infant undernutrition, inadequate care, and disadvantaged socioeconomic conditions in the first few years of life may cause the brain to fall short of its full potentials, which can be consequential for later-life cognitive aging (Barker, 2004; Borenstein & Mortimer, 2016a, pp. 121–151). Moreover, the *accumulation of risk model* posits that the exposures in early life may have a cumulative effect across the life course if the brain becomes vulnerable or weakened in keeping up with the accumulated damages (Kuh et al., 2003). In other words, exposure to adverse early-life circumstances may result in a faster rate of brain functioning loss, especially in later life. Finally, the *chains of risk model* argues that the exposures are linked across the life course (Kuh et al., 2003). One exposure in the early stage of life may lead to another exposure in later life, hence resulting in varying patterns of cognitive aging. Childhood SES, health and social environment, for instance, may determine the level of schooling, the patterns of socialization, and the extent to which individuals are involved in cognitively stimulating activities, which consequently change cognitive reserve and the progression of cognitive aging (Borenstein & Mortimer, 2016a, pp. 121–151; Foverskov et al., 2018; Glewwe & Miguel, 2007).

The transition from normal cognitive functioning to cognitive impairment can be slowed with better understanding of risk factors and their mechanisms. Particularly, promoting interventions targeting key social and environmental factors across the lifespan may increase individuals’ resilience to brain pathologies and therefore reduce vulnerability to cognitive impairment and dementia (Borenstein & Mortimer, 2016b, pp. 49–53; Stern, 2012). Given the long preclinical stages of the disorder, understanding early-life risk factors for cognitive aging is especially pivotal to delaying the disease progression and alleviating burdens of an aging society (Borenstein & Mortimer, 2016c, pp. 335–346; Sayer & Gill, 2016). However, few studies explicitly examine early-life determinants of cognitive aging, among which most focus on childhood SES and health factors (Fors et al., 2009; Kaplan et al., 2001; Luo & Waite, 2005), whereas investigations on other early-life factors, such as neighborhood social environments, are very limited (Wu et al., 2015). In addition, previous work tends to investigate a single early-life factor, while evidence that simultaneously considers a comprehensive set of circumstances are largely absent. Estimation biases may reduce after accounting for other relevant early-life factors (Borenstein & Mortimer, 2016a, pp. 121–151).

Moreover, prior research rarely distinguishes between the underlying impacts of early-life circumstances on two distinctive components of cognitive aging, i.e., the level of deficit and the rate of decline. Since level and rate may have different implications, this limits our understanding of cognitive aging process. In particular, the rate of cognitive decline often signals to individuals their potential cognitive problems that may promote timely diagnosis and treatment, while the level of cognitive deficit often determines the risk of being assessed cognitive impaired or even demented. The few studies that link childhood circumstances with later-life cognitive trajectories often provide inconsistent evidence: some shows that adverse childhood circumstances can lead to higher rates of cognitive decline (Brown, 2010; Marden et al., 2017; Melrose et al., 2015; Steptoe & Zaninotto, 2020), while others offer contradictory evidence (Barnes et al., 2012; Everson-Rose, 2003). Therefore, research on adverse childhood circumstances and cognitive aging is inconclusive.

To fill the gaps, this paper investigates the long-term effects of a wide spectrum of childhood circumstances on the trajectories of cognitive aging. Using three waves of the China Health and Retirement Longitudinal Survey (CHARLS 2011, 2013, 2015) and the CHARLS Life History Survey (2014), we characterize the varying cognitive aging patterns through four aspects of childhood circumstances: SES, neighborhood social environment, social relationships, and health conditions. Specifically, applying a linear mixed effect model to individuals’ trajectories of cognitive outcomes, we separate the baseline level of cognitive deficit from the rate of cognitive decline and respectively examine their associations with childhood circumstances.

As suggested by prior literature, these four domains of childhood factors may influence cognitive aging through multiple pathways. For example, childhood SES (Kaplan et al., 2001; Marden et al., 2017), neighborhood social environment (Wu et al., 2015), social relationships (Chan et al., 2019; Crosnoe, 2000) and health conditions (Kobayashi et al., 2017; Nguyen et al., 2008; Zhang et al., 2010) have profound effects on early-life brain development, which contribute to the initial cognitive reserve and vulnerability to brain pathologies. The four childhood circumstances may also determine the completion of formal education, social status, and health or health behaviors in adulthood (Borenstein & Mortimer, 2016a, pp. 121–151; Chetty et al., 2016; Fletcher et al., 2020; Glewwe & Miguel, 2007; Luo & Waite, 2005), which in turn shape varying patterns of cognitive aging. Further, childhood SES, neighborhood social environment, and social relationships may also be linked to social support and connections in adulthood (Crosnoe, 2000), which play an important role for cognitive functioning in old age (Bassuk et al., 1999; Borenstein & Mortimer, 2016d, pp. 281–290). Therefore, this study first tests the hypothesis that exposure to more adverse childhood circumstances is associated with faster cognitive aging. We then explore heterogeneous effects across gender, education, and rural/urban status. Finally, in light of the mechanisms discussed above, we examine the extent to which the effects are mediated through main pathways, including education, later-life family wealth, health and health behaviors, and social engagements.

This study contributes to the literature in three major aspects. First, the richness of life history data allows us to link, to our knowledge, the most comprehensive set of childhood circumstances with later life cognitive function. Second, we examine the long-term impacts of childhood circumstances on two distinctive dimensions of cognitive aging, i.e., the level of cognitive deficit and the rate of cognitive decline, which offers novel evidence on their relationships. Third, we underscore the importance of social relationships (e.g., childhood friendship and relationships with parents) and neighborhood social environments (e.g., neighborhood safety and cohesion) on cognitive deficit, which, to our knowledge, have not been thoroughly investigated in previous studies.

## 2 Data sets and methods

### 2.1 Data sources and analytical sample

Our analytical data are mainly obtained from the China Health and Retirement Longitudinal Study (CHARLS) conducted in 2011 (national baseline), 2013 (Wave 2 follow-up), 2014 (Life History Survey), and 2015 (Wave 3 follow-up), which collects a high quality and nationally representative sample of Chinese residents age 45 and older (Zhao et al., 2014). In addition, some key background characteristics controlled for in our analysis, such as age, education, and marital status, are extracted from Harmonized CHARLS, which integrates and validates the data from all four surveys (Beaumaster et al., 2018). The details of data sampling, collection, administration, as well as the obtainment of ethical approval and informed consent are presented in Appendix B.

We restrict our analysis to respondents aged 45 and older at baseline who have all three waves of cognitive test results, to ensure the validity of longitudinal cognitive measures. After excluding the illegible responses, 9109 respondents are used to model and decompose the individual trajectories of cognitive aging; they contribute a total of 27,327 observations in our analytical model (n = 9109 study samples × 3 time points). Of the 9109 respondents, 6700 participants have complete life history data and therefore are used to examine the association between childhood circumstances and two components of cognitive aging. We also check the balance of childhood characteristics between our study sample (n = 6700) and the sample with complete cognitive test results but incomplete life history data (n = 2409). As shown in Appendix Table A1, our study sample tends to be exposed to better childhood circumstances, as measured by parental education, neighborhood social environment, friendship, and health conditions. While potential selection of respondents experiencing adverse childhood circumstances and worse cognitive aging is unlikely, and therefore our concern over overestimated effects may be mitigated, we should still interpret our results with caution in consideration of these unbalanced factors.

### 2.2 Measures of childhood circumstances

Rich information about family history, health history, and other childhood environments is drawn from the CHARLS life history survey. Four domains of childhood circumstances, i.e., childhood SES, neighborhood social environment, social relationships, and health conditions, are considered, and objective measures for each domain are selected to ensure accuracy.

First, parental education, parental work status, and architecture type of the first residence are included to measure childhood SES. Among them, architecture type of the first residence is used as an objective measure of family economic and financial status (Ghawi et al., 2015; Zhao et al., 2020). Relative to the self-reported status collected in CHARLS, the housing characteristics (i.e., architecture type) has the advantage of objectivity and accuracy, which has been increasingly used in recent studies and recognized as a good indicator of individuals' SES (Ghawi et al., 2015; Juhn et al., 2011). Secondly, neighborhood safety and neighborhood cohesion are used to measure the childhood neighborhood social environments, which could also be important for individuals’ long-term cognitive development (Chan et al., 2019; Crosnoe, 2000; Wu et al., 2015; Yen et al., 2009). Third, childhood social relationships are captured by two measures: childhood friendship, childhood relationships with parents. Childhood friendship is measured by how often the respondent had a group of friends that he/she felt comfortable spending time with, which reflects the social supports and connections individuals had during the childhood. Childhood relationships with parents are intended to measure the level of family supports that individuals perceived (Borenstein & Mortimer, 2016d, pp. 281–290). Finally, childhood self-rated health, experience of serious illness, and experience of hospitalization are used to indicate childhood health status, and vaccination history and food deprivation during 0–5 years old are included as measures of childhood health resources (Kobayashi et al., 2017; Zhang et al., 2010).

The descriptive statistics of these variables are shown in Table 1; and additional details are presented in Appendix Table C1, which includes the original questions asked in the surveys and the construction and conceptualization of the variables.

**Table 1:** Descriptive statistics of childhood circumstances.

Childhood Circumstances	Level (%)
1. Childhood socioeconomic status	
Education of father	1. Illiterate (57.85); 2. Elementary school and below (34.49); 3. Middle school and above (7.66)
Education of mother	1. Illiterate (88.99); 2. Elementary school and below (9.57); 3. Middle school and above (1.45)
Work status of father	1. None or limited working (3.25); 2. Full-time farming work (78.39); 3. Full-time non-agricultural work (18.36)
Work status of mother	1. None or limited working (15.85); 2. Full-time farming work (79.81); 3. Full-time non-agricultural work (4.34)
Architecture type of first residence house	1. Concrete structure (11.55); 2. Adobe house (61.64); 3. Wood house/thatched houses (18.49); 4. Cave/Mongolian yurt/boat house/others (8.31)
2. Childhood neighborhood social environments	
Neighborhood safety	1. Very safe (50.22); 2. Somewhat safe (42.21); 3. Not very safe (5.54); 4. Not safe at all (2.03)
Neighborhood cohesion	1. Very close-knit (44.18); 2. Somewhat close-knit (51.91); 3. Not very close-knit (3.24); 4. Not close-knit at all (0.67)
3. Childhood social relationships	
Friendship	1. Often have a group of friends playing (65.54); 2. Sometimes (13.52); 3. Not very often (8.54); 4. Never (12.40)
Relationship with father	1. Fair/poor (19.72); 2. Good (80.28)
Relationship with mother	1. Fair/poor (17.25); 2. Good (82.75)
4. Childhood health conditions (before 15 years old)	
Relative health status compared to peers	1. Healthier (36.40); 2. about average (52.15); 3. Less Healthy (11.45)
Ever confined to bed more than one month	1. No (94.82); 2. Yes (5.18)
Ever hospitalized	1. No (98.13); 2. Yes (1.87)
Ever receive any vaccinations	1. No (13.76); 2. Yes (86.24)
Not enough food during 0–5 years old	1. No (65.01); 2. Yes (34.99)

*Notes:* N = 6700 individuals. First column shows the variable names and categories; and the second column shows the descriptive statistics of the childhood circumstances. The definition, construction and conceptualization of these variables are further presented in Appendix Table C1.

### 2.3 Measures of cognitive deficit

Cognitive deficit is assessed by five cognitive tests measured in the CHARLS baseline and two follow-up surveys: immediate word recall, delayed word recall, serial 7s (correctly subtracting 7 from the prior number), date naming (correctly reporting today's date), and picture drawing. Among them, immediate and delayed word recall tests, are used to assess individuals' short-term and long-term memory, whereas serial 7's test, date naming, and picture drawing are designed to assess the respondents' ability to perform mathematical tasks, orientation, and mental intactness. All the five cognitive tests are conducted by interviewers who are trained with a standard and stringent protocol. (Zhao et al., 2020, 2014). These tests have also been recognized as valid measures for cognition (Herzog & Wallace, 1997; Zhao et al., 2020), and the details related to the cognitive tests in CHARLS can be found in Appendix D.

As our goal is to examine the cognitive aging process, we sum all these test results to form a composite score (i.e., global cognitive function; range 0–30) and reverse-code to make it more interpretable, i.e., a greater value for the level of cognitive deficit or the rate of cognitive decline indicates a severer stage of cognitive aging (Xu et al., 2015). This composite measure has been shown to have a strong relationship with defining cognitive impairment, thus is a good measure of respondents’ overall cognitive functioning (Herzog & Wallace, 1997; Langa et al., 2008). The distributions of cognitive deficits in our study sample are shown in Appendix Figure A1.

**Fig. 1:**
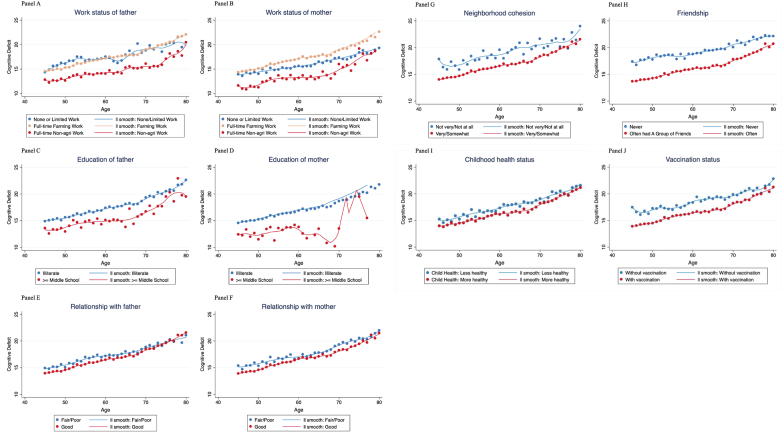
Course of cognitive aging with diverse childhood circumstances. *Notes:* Panel A–J illustrate the diverse course of cognitive aging from age 45 to age 80 with different childhood circumstances, including parental education, parental work status, childhood relationships, childhood neighborhood social environment, and childhood health conditions. The X axis denotes the respondents’ age when their cognitions were assessed. As cognition function is measured longitudinally in CHARLS, each individual may contribute more than one observation to the trend, and his/her cognitive function may reflect in more than one age group depending on the exact time of cognitive assessment. The plotted points in each panel, thus, represent the average level of cognitive deficit, for the ones with particular ages when the cognitive test was conducted, and with particular childhood circumstances. Cognitive deficit is defined as the reversed summary score of five cognitive tests, with higher value indicating greater cognitive deficit; age is specified as the age when the cognitive tests were conducted. All the regression lines are fitted using local linear smoothing.

To characterize the varying patterns of cognitive aging related to childhood circumstances, we plot the average trends of cognitive deficit by childhood circumstance. As shown in Fig. 1, cognition gaps exist between cohorts of diverse childhood circumstances. Those with better childhood circumstances generally have a lower level of cognitive deficit. The differences persist for all age groups from age 45 to age 80. Moreover, there are large variations across childhood characteristics. Some of the gaps are larger, such as work status of parents and childhood friendship, while others seem smaller, such as relationship with parents.

### 2.4 Other variables

In addition to the childhood circumstances and cognitive outcomes, we include a set of covariates and mediators to adjust for their associations with both the exposures and outcomes. Specifically, a number of covariates are controlled for in our main analysis, including baseline age, gender, education, hukou status (rural/urban), marital status, log income, and the number of chronic diseases.

In addition, as suggested by the existing literature, several important mediators could potentially link childhood circumstances with later-life cognitive aging, including formal education, later-life family wealth, health and health behaviors, and social engagements. These factors are further examined and compared in the mediation analysis.

The definition and construction of these variables are presented in Appendix Table C2.

### 2.5 Empirical strategy

The descriptive analysis only captures the average population trend, hence in our study, we model individual cognitive aging trajectories to shed light on within-subject pattern of cognitive decline.

Linear mixed-effect model (LMM) is utilized to model the individual development of health outcomes by adjusting for the correlations of the repeated measures within one subject (Burton-Jeangros et al., 2015; Laird & Ware, 1982). An emerging strand of literature in cognitive science have used the linear mixed-effect model to investigate the trajectory of cognitive aging (Hall et al., 2000; Hout et al., 2015; Wilson et al., 2011). In our study, the model used can be specified as,

(1) $$Y_{it}=\gamma_{0}+\gamma_{1}Time_{it}+\gamma_{2}X_{i}+\gamma_{3}X_{i}\;Time_{it}+\mu_{0i}+\mu_{1i}Time_{it}+\varepsilon_{it}$$

where $Y_{it}$ is the composite score of cognitive deficit measured for individual i at time t, and $\gamma_{0}$ and $\gamma_{1}$ are the fixed intercept and fixed slope for the study population; $X_{i}$ is the covariates matrix controlled in our model, including baseline age, gender and education level (Wilson et al., 2011); interaction term $X_{i}\times Time_{\text{it}}$ is added into the model to adjust for the fixed impact of covariates on the slope. Thus, $\gamma_{2}$ and $\gamma_{3}$ respectively represent the fixed impact of covariates on baseline level and slope. Finally, $\mu_{0i}$ and $\mu_{1i}$ represent the random intercepts and random slopes for the individual i, which capture the individual deviations from the central values of intercept (i.e., $\gamma_{0}+\gamma_{2}X_{i}$) and slope (i.e., $\gamma_{1}+\gamma_{3}X_{i}$).

Based on these coefficient estimates, we calculate the baseline level of cognitive deficit and rate of cognitive decline for each individual i as a combination of group fixed effect, $\gamma_{0}+\gamma_{2}X_{i}$ and $\gamma_{1}+\gamma_{3}X_{i}$, and individual random effect, $\mu_{0i}$ and $\mu_{1i}$. (Belsky et al., 2015; Burton-Jeangros et al., 2015),

(2) $$L_{i}=\gamma_{0}+\gamma_{2}X_{i}+\mu_{0i}$$

(3) $$R_{i}=\gamma_{1}+\gamma_{3}X_{i}+\mu_{1i}$$

After obtaining individual level of cognitive deficit, $L_{i}$, and rate of decline, $R_{i}$, we use linear regressions to study the association of childhood characteristics with the level of cognitive deficit and the rate of decline. We do not directly include our comprehensive set of childhood characteristics and their time interaction terms in the linear mixed-effect model to avoid overparameterizing or mis-specifying the model (Bolker et al., 2009; Harrison et al., 2018). The regression equation is illustrated as follows,

(4) $$Y_{i}=\alpha +\beta \cdot EarlyLife_{i}+\delta \cdot X_{i}+\varepsilon_{i}$$

where $Y_{i}$ is the outcome of interest, representing either the level of cognitive deficit, $L_{i}$, or the rate of decline, $R_{i}$, of individual i. $EarlyLife_{i}$ include four domains of childhood circumstances, including childhood SES, neighborhood social environment, social relationships, and health conditions. $X_{i}$ contains a set of covariates, including baseline age, gender, education level, rural/urban hukou status, marital status, log income and number of chronic diseases. Each domain of childhood circumstances is added subsequently into linear regression, from Model 1 with only childhood SES to Model 4 with all four domains of childhood circumstances, to check robustness of our findings. Among them, Model 4 is our preferred model specification with complete sets of childhood factors. Because the long-term health impacts could vary across gender (Lei et al., 2012), baseline rural/urban status (Zhang et al., 2017), and education level (Foverskov et al., 2018), we also explore the heterogeneity of the effects across these subgroups and test the statistical significance of the differences between two groups following the Chow test (Chow, 1960).

In addition to our main regression analyses, we conduct a set of mediation analyses to provide suggestive evidence on the mechanisms of the effects following the Difference Method (VanderWeele, 2016). In particular, we examine whether including potential mediators (e.g., education, social engagements) in the regression model would attenuate the exposure estimates. If the coefficient estimates of particular childhood circumstances reduce markedly after accounting for potential mediators, this would be a signal of mediation that can explain some of the effects of the childhood circumstances on cognitive aging and may corroborate certain pathways (VanderWeele, 2016).

All regression models are weighted using individual sample weights, with household and individual non-response adjustment. Standard errors are clustered at urban/rural communities to account for correlation within clusters. Detailed analytical procedure is illustrated in Appendix Figure A2. All the data are analyzed using Stata 16.1.

## 3 Results

Decomposing cognitive aging into the level of cognitive deficit and the rate of cognitive decline, the summary statistics of our model estimates are shown in Table 2. Using the sample with complete cognition data (N = 9109), we obtain the average baseline level of 15.77, with the level estimates $L_{i}$ ranging from 6.43 to 25.64. The average rate of cognitive decline $R_{i}$ is 0.23, with maximum value of 1.00. For the sample with complete cognition and life history data (N = 6700), the summary statistics of these two measures are similar to the estimates using the full sample, reducing the potential concern over selection bias. A scatterplot of the level and rate estimates is shown in Appendix Figure A3. We next present the results on childhood circumstances and two components of cognitive aging, respectively.

**Table 2:** Descriptive statistics of baseline level of cognitive deficit and rate of cognitive decline estimates.

Cognitive measures	N	Mean	SD	Min	Max
Level *L*_*i*_	9109	15.77	3.41	6.43	25.64
Rate of Decline *R*_*i*_	9109	0.23	0.20	−0.34	1.00
Level *L*_*i*_ (with complete life history data)	6700	15.60	3.35	6.43	25.33
Rate of Decline *R*_*i*_ (with complete life history data)	6700	0.22	0.19	−0.34	1.00

*Notes:* Individual level *L*_*i*_ and rate *R*_*i*_ are estimated using linear mixed-effect model. Row 1 and row 2 are the summary statistics of sample with three waves of cognitive tests (N = 9109). Row 3 and row 4 are the summary statistics of subsample with three waves of cognitive tests and complete life history data (N = 6700).

### 3.1 Association between childhood circumstances and the level of cognitive deficit

Table 3 reports the linear regression estimates with different model specifications. In the model with only childhood SES and covariates (i.e., Column 1), father's education, parental work status, and the first residence architecture type are significantly associated with baseline level of deficit. Although the estimates slightly decline as we add more domains of variables into the regressions (i.e., Column 3, 5, and 7), they remain statistically significant.

**Table 3:** Regression results of the association of childhood circumstances with the level of cognitive deficit (intercept) and the rate of cognitive decline (slope).

	Model 1		Model 2		Model 3		Model 4	
	(1)	(2)	(3)	(4)	(5)	(6)	(7)	(8)
	Level	Rate	Level	Rate	Level	Rate	Level	Rate
Education of father (Ref. Illiterate)								
Elementary school and below	−0.474***	−0.018***	−0.475***	−0.018***	−0.454***	−0.017***	−0.452***	−0.017***
	(<0.001)	(<0.001)	(<0.001)	(<0.001)	(<0.001)	(<0.001)	(<0.001)	(<0.001)
Middle school and above	−0.388**	−0.011*	−0.377**	−0.011*	−0.366**	−0.011	−0.356**	−0.010
	(0.006)	(0.044)	(0.006)	(0.045)	(0.007)	(0.056)	(0.007)	(0.056)
Education of mother (Ref. Illiterate)								
Elementary school and below	−0.217	−0.008	−0.222	−0.008	−0.209	−0.008	−0.197	−0.007
	(0.059)	(0.075)	(0.054)	(0.066)	(0.065)	(0.071)	(0.081)	(0.090)
Middle school and above	−0.959	−0.039*	−0.929	−0.039*	−0.898	−0.037*	−0.923	−0.038*
	(0.062)	(0.018)	(0.064)	(0.019)	(0.077)	(0.025)	(0.060)	(0.016)
Work status of father (Ref. None/limited)								
Full-time farming work (Farther)	−0.373	−0.018*	−0.341	−0.017*	−0.343	−0.017*	−0.326	−0.016*
	(0.062)	(0.026)	(0.088)	(0.038)	(0.084)	(0.034)	(0.094)	(0.037)
Full-time non-agricultural work	−0.450*	−0.020*	−0.426*	−0.019*	−0.422*	−0.019*	−0.380	−0.017*
	(0.034)	(0.023)	(0.043)	(0.029)	(0.043)	(0.029)	(0.062)	(0.044)
Work status of mother (Ref. None/limited)								
Full-time farming work (Mother)	0.208*	0.005	0.201*	0.005	0.195*	0.004	0.191*	0.004
	(0.024)	(0.248)	(0.029)	(0.270)	(0.032)	(0.290)	(0.034)	(0.293)
Full-time non-agricultural work	−0.301	−0.012	−0.311	−0.012	−0.303	−0.012	−0.300	−0.011
	(0.132)	(0.125)	(0.126)	(0.122)	(0.127)	(0.121)	(0.131)	(0.128)
Architecture type (Ref. concrete structure)								
Adobe house	0.395**	0.016**	0.391**	0.016**	0.388**	0.015**	0.375**	0.015**
	(0.001)	(0.005)	(0.001)	(0.006)	(0.001)	(0.005)	(0.002)	(0.007)
Wood/thatched house	0.538***	0.022***	0.523***	0.021***	0.515***	0.021***	0.501***	0.020***
	(<0.001)	(<0.001)	(<0.001)	(<0.001)	(<0.001)	(<0.001)	(<0.001)	(<0.001)
Cave/Mongolian yurt/boat house/others	0.248	0.007	0.239	0.007	0.226	0.006	0.218	0.006
	(0.163)	(0.370)	(0.179)	(0.384)	(0.183)	(0.412)	(0.193)	(0.433)
Neighborhood safety (Ref. very safe)								
Somewhat safe			−0.061	−0.002	−0.061	−0.002	−0.068	−0.002
			(0.349)	(0.622)	(0.344)	(0.610)	(0.290)	(0.532)
Not very safe			−0.000	0.003	−0.071	0.000	−0.096	−0.001
			(0.999)	(0.589)	(0.655)	(0.941)	(0.550)	(0.867)
Not safe at all			0.657**	0.020*	0.665**	0.020*	0.626**	0.018
			(0.003)	(0.047)	(0.003)	(0.043)	(0.005)	(0.065)
Neighborhood cohesion (Ref. very close)								
Somewhat close-knit			0.210**	0.003	0.139	0.001	0.123	−0.000
			(0.005)	(0.298)	(0.064)	(0.836)	(0.100)	(0.992)
Not very close-knit			0.462*	0.017	0.292	0.010	0.261	0.008
			(0.020)	(0.061)	(0.140)	(0.272)	(0.186)	(0.351)
Not close-knit at all			1.612***	0.054***	1.381***	0.046**	1.354***	0.044**
			(<0.001)	(<0.001)	(<0.001)	(0.003)	(<0.001)	(0.004)
Friendship (Ref. often)								
Sometimes					0.248*	0.010*	0.226*	0.009*
					(0.010)	(0.014)	(0.019)	(0.025)
Not very often					0.352**	0.016**	0.333**	0.014**
					(0.003)	(0.001)	(0.005)	(0.003)
Never					0.772***	0.030***	0.747***	0.029***
					(<0.001)	(<0.001)	(<0.001)	(<0.001)
Relationship with mother (Ref. Fair/Poor)								
Good (Mother)					−0.154	−0.012**	−0.139	−0.011*
					(0.177)	(0.010)	(0.221)	(0.015)
Relationship with father (Ref. Fair/Poor)								
Good (Father)					0.002	0.007	0.010	0.008
					(0.988)	(0.095)	(0.928)	(0.085)
Relative Health Status (Ref. Healthier)								
About average							0.209**	0.009**
							(0.003)	(0.001)
Less healthy							0.141	0.004
							(0.284)	(0.473)
Confined to bed (Ref. No)								
Yes							−0.082	0.001
							(0.647)	(0.860)
Hospitalized (Ref. No)								
Yes							0.269	0.011
							(0.328)	(0.312)
Ever receive vaccinations (Ref. No)								
Yes							−0.248*	−0.014***
							(0.011)	(<0.001)
Not enough food during 0–5 (Ref. No)								
Yes							0.172*	0.009**
							(0.022)	(0.009)
								
Observations	6700	6700	6700	6700	6700	6700	6700	6700
R-squared	0.547	0.767	0.550	0.768	0.556	0.771	0.558	0.773
Covariates	YES	YES	YES	YES	YES	YES	YES	YES

*Notes:* N = 6700 observations. Standard errors are clustered at community level. Covariates are controlled in all four models, including age, gender, education, hukou status (rural/urban), marital status, log income and number of chronic diseases. Regressions are weighted at individual level with household and individual non-response adjustment. P-values are shown in parentheses. Statistical significance: ***p < 0.001, **p < 0.01, *p < 0.05.

In our preferred model with complete sets of childhood circumstances (i.e., Column 7), we find a negative association between father's education and the level of cognitive deficit. In addition, compared to those whose mothers had no full-time job, people whose mothers worked in full-time farming, often indicating disadvantaged family SES or limited time with children, show higher level of cognitive deficit (Browning et al., 2014). Furthermore, people living in more inferior residence during childhood show a significantly greater level of cognitive deficit.

Neighborhood cohesion and safety are found to be strongly associated with later life cognitive deficit. People who lived in a less close-knit and unsafe community show a significantly higher level of cognitive deficit. We also find a strong protective effect of a good childhood friendship on cognitive aging. However, no significant association between childhood relationships with parents and level of cognitive deficit is observed.

Moreover, poor childhood health status is significantly associated with a higher level of cognitive deficit; people with insufficient vaccination and nutrition in early childhood (0–5 years old) also show significantly greater cognitive deficits in later life.

To examine robustness of the results, we also conduct linear regressions based on each of the three waves of cognitive deficit scores. As shown in Table A2, these results (i.e., Column 1–3) as well as those obtained based on the pooled three waves of data (i.e., Column 4) are consistent with the results on level of cognitive deficit obtained from LMM (i.e., Column 5, which repeats Column 7 of Table 3 for ease of comparisons).

**Fig. 2:**
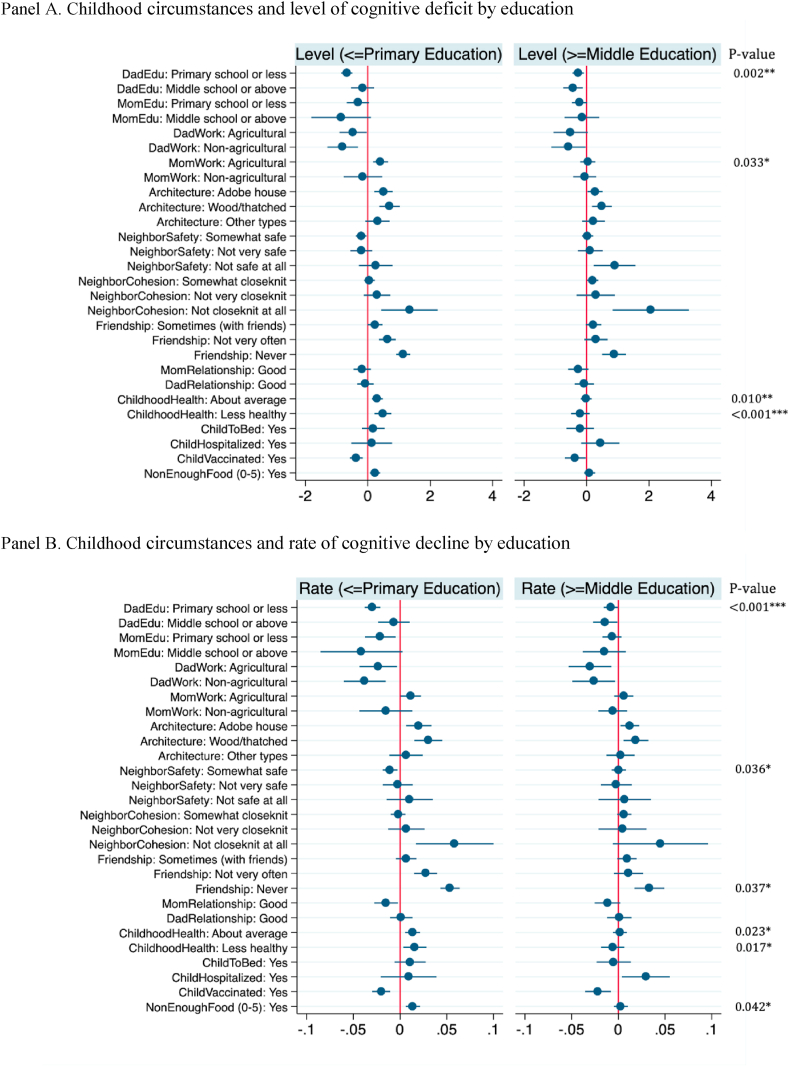
The effects of childhood circumstances on cognitive aging among people with lower and higher education. Panel A. Childhood circumstances and level of cognitive deficit by education.Panel B. Childhood circumstances and rate of cognitive decline by education. Notes: Coefficient plots of the childhood circumstances on level of cognitive deficit (Panel A) and rate of decline (Panel B) among people with lower (primary school or below) and higher education level (middle school or above). The cross-equation test is conducted respectively to examine the statistical difference between the coefficients in two linear regressions. P-value is calculated based on Chow/Wald test, showing at the rightmost side of each panel (Only significant results are illustrated, whereas other estimates are available upon request. Statistical significance: ***p < 0.001, **p < 0.01, *p < 0.05).

Lastly, we explore heterogeneous effects. In particular, we apply our full model (i.e., Model 4) to the subsamples of low versus high levels of education, males versus females, and rural versus urban hukou status at baseline. The coefficient estimates are respectively plotted in Fig. 2, Appendix Figure A4 and Figure A5. As shown in Fig. 2, significantly larger effects are found for the subsample with less education (i.e., primary school or below) than that with more education in the effects of father's education, mother's work status, and childhood health status on the level of cognitive deficit. By contrast, the sizes of the effects are largely similar between male and female subsamples (Figure A4). Father's work status and childhood health status show smaller effects for urban than rural samples (Figure A5), suggesting the potential role of social welfare benefits.

### 3.2 Association between childhood circumstances and the rate of cognitive decline

The estimates of childhood circumstances on rate of cognitive decline are shown in Columns 2, 4, 6, and 8 in Table 3. In our full model (Column 8, Table 3), people who report greater father's education and work status, higher mother's education, better family residence, greater neighborhood cohesion, more friendship, better relationship with mother, better health status, more vaccination, and better nutrition are found to have a significantly lower rate of cognitive decline.

Some salient differences are identified comparing the relationships between childhood circumstances and the two components of cognitive aging. Specifically, unlike the results for level of cognitive deficit (Column 7, Table 3), no significant association is observed between childhood neighborhood safety and rate of cognitive decline. Moreover, having a good relationship with mother are significantly associated with a lower rate of cognitive decline but not the level of cognitive deficit. Nonetheless, childhood neighborhood cohesion, friendship, health status, vaccination, and nutrition show significant association with both components of cognitive aging. In particular, the gradient of association between childhood friendship and cognitive aging indicates a strong protective effect of friendship.

To enable more meaningful comparisons between the level of cognitive deficit and the rate of cognitive decline, we standardize the coefficient estimates of our full model (i.e., Model 4, Table 3) in standard deviations (SDs) and presented the effect size in Table A2, Columns 7 and 8. Our results indicate that, one SD change in childhood circumstances mostly have larger effects on the level of cognitive deficit than those on the rate of cognitive decline, in terms of changes in SD. Nevertheless, the effect size of relationship with parents is larger on the rate of decline than on baseline level.

Finally, similar to our findings on cognitive level, the impacts of father's education, childhood health status and malnutrition, neighborhood safety, childhood friendship are larger on the rate of cognitive decline for the less educated subsample; and the impact of father's work status is more salient for rural than urban samples. No significant difference is found between male and female samples.

### 3.3 The mediation effects of adult-life and later-life factors

The chains of risk model suggests that childhood circumstances may affect cognitive aging through adulthood exposures, which enable a set of important pathways. To offer suggestive evidence, we examine the roles of educational attainment, later-life family wealth, health and health behaviors, and social engagements in the relationships between childhood circumstances and cognitive aging. We cumulatively include these factors into the model to test if they attenuate the effects of some or all aspects of childhood circumstances on cognitive aging. In addition, because childhood neighborhood social environment and social relationships are fundamental in shaping patterns of socialization, which may influence the onset of dementia or cognitive decline (Borenstein & Mortimer, 2016d, pp. 281–290), we explore the extent to which their effects on cognitive aging can be mediated by later-life social engagements.

In Appendix Table A3, we first compare the exposure estimates of the regressions with versus those without controlling for education. Adding education into the model substantially attenuate the coefficient estimates of all childhood circumstances. The reductions in the size of the coefficients are 23–53% for childhood SES, 19–31% for neighborhood social environment, 9–60% for social relationships, and 29–58% for health conditions. These findings thus imply that the effects of childhood circumstances on cognitive aging can be mediated by education. Moreover, in Appendix Table A4, we cumulatively control for later-life family wealth, health and health behaviors, and social engagements. Results show that including family wealth, health and health behaviors has little impact on the estimates of childhood exposure, while controlling for social engagements shrinks the coefficients of neighborhood cohesion and friendship, though the size of mediation effect is smaller than that led by education. Therefore, adulthood social engagement is likely an underlying pathway through which neighborhood cohesion and friendship in childhood influence cognitive aging. Overall, education seems the most important mediator.

## 4 Conclusions and discussion

Childhood family socioeconomic conditions, health, community environment, and relationships can lead to increased vulnerability to the cognitive aging process later in life. This study offer novel evidence on the long-term relationship between a comprehensive set of these childhood circumstances and cognitive aging (Luo & Waite, 2005; Zhang et al., 2008). We also advance the literature by offering novel evidence with longitudinal data and a mixed effect model to distinguish key components of cognitive aging (Fors et al., 2009; Kaplan et al., 2001). Our finding suggests varying effects of childhood circumstances on components of cognitive aging, including the level of cognitive deficit and the rate of cognitive decline. In particular, one SD change in childhood circumstances often have larger effects on the level of cognitive deficit than on the rate of cognitive decline, except for the relationship with parents. Finally, we also offer novel evidence on own educational attainment mediating the effect of a wide spectrum of adverse childhood circumstances.

First, we show that exposure to adverse childhood SES or health conditions may worsen both components of cognitive aging. Though the size and significance of the effects vary by factors, this pattern may reflect two important pathways. On the one hand, father's education, family housing status, and child health conditions may have profound effects on children's cognitive development, reserve and cognitive aging (Borenstein & Mortimer, 2016a, pp. 121–151). Father's education largely affects labor supply and determines the resources that a family can invest in children (Browning et al., 2014); and family housing and child health conditions (especially vaccination and nutritional status) to large extent reflect the family resources and society support available. Disadvantaged SES in early life hence may greatly limit the level of resources provided to children, impede individuals' healthy brain development, and in turn expose them to adverse brain pathologies and functioning loss in later life (Noble et al., 2015; Staff, 2012). On the other hand, disadvantaged early-life SES and health conditions may also affect later-life cognitive deficit and decline through a chain of adult-life exposures, such as education, employment, health conditions, and health behaviors (Borenstein & Mortimer, 2016a, pp. 121–151), where education seems a more important channel as indicated in this study. For example, this study show that mother's education had significant effects on *both* components of cognitive aging without adjusting for own education, but *only* affecting rate of cognitive decline after adjusting for own education.

Second, we find that the relationship with mother can buffer against cognitive decline in later life; whereas the relationship with father cannot. This pattern can be explained by the different roles that father and mother play at home, which contribute differently to children's cognitive development. Literature suggests that parents collectively allocate their time to labor market and investments in children given their resources and preferences (Blundell et al., 2005). With more human capital, fathers tend to spend more time on the labor market, while mothers invest more time in children. Hence, as the major caregiver at home, mothers tend to spend more time in educating and interacting with children than fathers do (Browning et al., 2014). Mothers are also more likely to be the main decision maker for children's health inputs and education (Attanasio et al., 2012). Children with better relationships with mothers in early life thus are more likely to receive adequate care, education, and intellectual stimulation at home, and are more resilient to brain pathologies in later life (Murray et al., 2012; Noble et al., 2015).

Finally, we reveal how childhood friendship and neighborhood social environment can be associated with later life cognitive aging. In particular, we find that childhood friendship and neighborhood cohesion have strong protective impacts on both dimensions of cognitive aging. Three potential pathways may account for the relationships. First, childhood friendship and neighborhood cohesion represent the social support and connections individuals have that may benefit cognitive health in terms of initial level of reserve or vulnerability to brain pathologies (Borenstein & Mortimer, 2016d, pp. 281–290). Second, these childhood factors, especially friendship, may influence educational attainment and health behaviors (Fletcher et al., 2020; Fletcher & Ross, 2018), which in turn impose effects on cognitive aging. Our mediation analysis shows that education account for a considerable part of the associations. Third, better childhood friendship and neighborhood cohesion may influence cognitive aging through more active social engagements in adulthood (Crosnoe, 2000), which helps build cognitive reserve or prevent functional loss in the life course (Bassuk et al., 1999). In comparison, neighborhood safety, i.e., another aspect of neighborhood social environment, is mainly linked to stress and other psychosocial factors with influence on initial cognitive function and reserve, while the pathway through which it affects later-life cognitive change is relatively limited (Wu et al., 2015). Consistently, this study finds a significant effect of neighborhood safety on the level of cognitive deficit but not on the cognitive decline, and the effect size for neighborhood safety is small.

Overall, our findings lend support to studies on life course cognitive health. First, growing evidence shows independent associations between childhood SES and later-life cognitive function (Fors et al., 2009; Kaplan et al., 2001; Luo & Waite, 2005; Marden et al., 2017; Nguyen et al., 2008) and cognitive decline (Brown, 2010; Marden et al., 2017; Melrose et al., 2015; Steptoe & Zaninotto, 2020). Second, existing literature have also demonstrated the important role of child health and nutrition in determining both components of cognitive aging (Kobayashi et al., 2017; Nguyen et al., 2008; Zhang et al., 2010). Third, though prior research mainly focuses on the impact of social environment on cognitive function but not on cognitive trajectories (see a systematic review Wu et al., 2015), cross-sectional studies show evident link between neighborhood safety and cognitive deficit (Wu et al., 2015; Yen et al., 2009). Fourth, while there is no direct evidence on the relationship between childhood friendship, social cohesion and cognitive aging, partly due to challenges in collecting life history data, our findings are supported by a strand of literature on later-life social cohesion, social networks and cognitive aging (Bassuk et al., 1999; Borenstein & Mortimer, 2016d, pp. 281–290; James et al., 2012). Emerging research on childhood social activities also corroborate our results (Chan et al., 2019).

Some limitations could impede the generalizability of this study. First, although cognitive deficit is longitudinally examined, only three waves of cognitive assessments are collected. Cognitive aging trajectories may be better modeled with longer follow-up waves. Second, as most of our childhood factors are self-reported, results may suffer from recall bias (Borenstein & Mortimer, 2016a, pp. 121–151), despite our intention to select more objective measures. Third, although the CHARLS survey respondents are randomly sampled and their information is collected following a well-administered process (Zhao et al., 2020, 2014), the sample with missing values in certain childhood circumstances or cognitive outcomes can be nonrandomly missing, which implies the existence of selection bias. For example, people with more disadvantaged childhood circumstances may have more difficulty understanding the questions to comply with the surveying process. Survival bias may select healthier older adults or those who experienced more favorable circumstances in early life. Hence, our findings should be interpreted with caution. Fourth, we offer initial evidence on associations between childhood circumstances and key components of cognitive aging. No causal relationship can be drawn at this stage. The underlying mechanisms require further examinations with causal study designs. Finally, future work will understand the mediating effects of life course factors other than education.

Despite these limitations, our study may have valuable policy implications. First, we have shown that a wide range of childhood circumstances could contribute to the early onset and progression of cognitive aging, even after controlling for education and other adult-life characteristics. This finding highlights the critical and persisting impacts childhood adversity may have across the life course. Hence, to delay pathologic evolution and promote healthy aging, it is important to intervene early in life by providing adequate social support and resources. Timely interventions during childhood would generate significant health benefits in the long term and relieve the burden of population aging. Second, though childhood circumstances may affect different dimensions of cognitive aging (deficits vs. trajectories) through different pathways, they share some common grounds that require targeted interventions. On the one hand, the adversity of childhood circumstances, such as low parental SES, food deprivation, and lack of vaccination, reflect the inadequacy of social and economic policies, emphasizing the significance of public investments in education, public health programs, and targeted transfer programs. On the other hand, the establishment of advantaged childhood circumstances require joint efforts from families and society. In particular, families and society should not only work together to provide sufficient resources for children, but also build a supporting environment that is beneficial for individuals’ health and social wellbeing, especially given the important roles of social cohesion and relationships revealed in this study. Finally, the large differences in childhood circumstances imply the needs for training and educational programs to narrow the gap in cognitive skills across contexts of different educational background to enhance comparability and accuracy of cognitive assessments. Improved cognitive assessments make the surveillance and early targeting of cognitive impairment and dementia more efficient.

## Funding

This study was funded by Yale Macmillan Center faculty research award, the U.S. PEPPER Center Scholar Award (P30AG021342), and two NIH/NIA grants (K01AG053408; R03AG048920).

## CRediT authorship contribution statement

**Zhuoer Lin:** participated in the study design, analyzed data, participated in the interpretation of results, drafted the manuscript, contributed to review the manuscript, All authors read and approved the final manuscript. **Xi Chen:** participated in the study design, participated in the interpretation of results, drafted the manuscript, contributed to review the manuscript.

## Declaration of competing interest

The authors declare that they have no conflict of interest.
