# Supplementary material for: Adverse childhood circumstances and cognitive function in middle-aged and older Chinese adults: Lower level or faster decline?
Source: SSM Popul Health. 2021 Mar 15;14:100767. doi: 10.1016/j.ssmph.2021.100767 (PMC8025052; doi:10.1016/j.ssmph.2021.100767)
Supplement: Multimedia component 1 [file mmc1.docx]

**Appendix A. Supplementary Results**

**Table A1.** Comparisons between our study sample and the sample with missing data

| Childhood Circumstances | Study Sample  (N=6,700) | Sample with Incomplete Life History Data  (N=2,409) | P-value |
| --- | --- | --- | --- |
|  | (1) | (2) | (3) |
| 1. Childhood socioeconomic status |  |  |  |
| Education of father |  |  |  |
| Illiterate | 57.85 | 61.60 | 0.039 |
| Elementary school and below | 34.49 | 31.39 |  |
| Middle school and above | 7.66 | 7.02 |  |
|  |  |  |  |
| Education of mother |  |  |  |
| Illiterate | 89.23 | 91.79 | 0.001 |
| Elementary school and below | 9.24 | 6.65 |  |
| Middle school and above | 1.53 | 1.56 |  |
|  |  |  |  |
| Work status of father |  |  |  |
| None or limited working | 3.25 | 4.35 | 0.075 |
| Full-time farming work | 78.39 | 78.31 |  |
| Full-time non-agricultural work | 18.36 | 17.34 |  |
|  |  |  |  |
| Work status of mother |  |  |  |
| None or limited working | 15.85 | 14.93 | 0.160 |
| Full-time farming work | 79.81 | 79.75 |  |
| Full-time non-agricultural work | 4.34 | 5.32 |  |
|  |  |  |  |
| Architecture type of first residence house |  |  |  |
| Concrete structure | 11.55 | 13.10 | 0.087 |
| Adobe house | 61.64 | 62.43 |  |
| Wood house/thatched houses | 18.49 | 17.04 |  |
| Cave/Mongolian yurt/boat house/others | 8.31 | 7.42 |  |
|  |  |  |  |
| 2. Childhood neighborhood social environments |  |  |  |
| Neighborhood safety |  |  |  |
| Very safe | 50.22 | 46.97 | <0.001 |
| Somewhat safe | 42.21 | 42.14 |  |
| Not very safe | 5.54 | 7.77 |  |
| Not safe at all | 2.03 | 1.69 |  |
|  |  |  |  |
| Neighborhood cohesion |  |  |  |
| Very close-knit | 44.18 | 39.97 | <0.001 |
| Somewhat close-knit | 51.91 | 53.87 |  |
| Not very close-knit | 3.24 | 4.46 |  |
| Not close-knit at all | 0.67 | 1.69 |  |
|  |  |  |  |
| 3. Childhood social relationships |  |  |  |
| Friendship |  |  |  |
| Often have a group of friends playing | 65.54 | 58.60 | <0.001 |
| Sometimes | 13.52 | 13.85 |  |
| Not very often | 8.54 | 10.91 |  |
| Never | 12.40 | 16.64 |  |
|  |  |  |  |
| Relationship with father |  |  |  |
| Fair/Poor | 19.72 | 21.24 | 0.184 |
| Good | 80.28 | 78.76 |  |
|  |  |  |  |
| Relationship with mother |  |  |  |
| Fair/Poor | 17.25 | 18.41 | 0.260 |
| Good | 82.75 | 81.59 |  |
|  |  |  |  |
| 4. Childhood health conditions (before 15 yrs old) |  |  |  |
| Relative health status compared to peers |  |  |  |
| Healthier | 36.40 | 33.67 | <0.001 |
| About average | 52.15 | 50.98 |  |
| Less Healthy | 11.45 | 15.35 |  |
|  |  |  |  |
| Ever confined to bed more than one month |  |  |  |
| No | 94.82 | 94.82 | 0.467 |
| Yes | 5.18 | 5.59 |  |
|  |  |  |  |
| Ever hospitalized |  |  |  |
| No | 98.13 | 98.14 | 0.998 |
| Yes | 1.87 | 1.86 |  |
|  |  |  |  |
| Ever receive any vaccinations |  |  |  |
| No | 13.76 | 17.51 | <0.001 |
| Yes | 86.24 | 82.49 |  |
|  |  |  |  |
| Not enough food during 0-5 years old |  |  |  |
| No | 65.01 | 61.08 | 0.002 |
| Yes | 34.99 | 38.92 |  |

*Notes:* Column 1 shows the distribution (in proportion) of life history variables among the sample included in our regression analysis (N=6,700), while Column 2 shows the distribution of the sample with complete cognitive tests, but incomplete life history data (N=2,409). Column 3 shows the p-value of Pearson chi2 test.

**Table A2.** Comparison of the regression results using the reversed cognitive test scores of three measured waves and the regression results using the levels and rates estimated from linear mixed effect model

|  | Cross-sectional | | | | LMM Decomposition | | Effect Size | |
| --- | --- | --- | --- | --- | --- | --- | --- | --- |
|  | (1) | (2) | (3) | (4) | (5) | (6) | (7) | (8) |
|  | Wave 2011 | Wave 2013 | Wave 2015 | Pooled Avg | Level | Rate | Level | Rate |
| Education of father (Ref. Illiterate) |  |  |  |  |  |  |  |  |
| Elementary school and below | -0.596*** | -0.671*** | -0.750*** | -0.672*** | -0.452*** | -0.017*** | -0.064 | -0.041 |
|  | (<0.001) | (<0.001) | (<0.001) | (<0.001) | (<0.001) | (<0.001) |  |  |
| Middle school and above | -0.495* | -0.713** | -0.374 | -0.527** | -0.356** | -0.010 | -0.029 | -0.015 |
|  | (0.040) | (0.003) | (0.145) | (0.007) | (0.007) | (0.056) |  |  |
| Education of mother (Ref. Illiterate) |  |  |  |  |  |  |  |  |
| Elementary school and below | -0.335 | -0.127 | -0.418* | -0.293 | -0.197 | -0.007 | -0.018 | -0.012 |
|  | (0.118) | (0.584) | (0.036) | (0.079) | (0.081) | (0.090) |  |  |
| Middle school and above | -1.296 | -0.866 | -1.967* | -1.377 | -0.923 | -0.038* | -0.039 | -0.028 |
|  | (0.165) | (0.146) | (0.016) | (0.058) | (0.060) | (0.016) |  |  |
| Work status of father (Ref. None/limited) |  |  |  |  |  |  |  |  |
| Full-time farming work (Farther) | -0.155 | -0.658* | -0.655 | -0.489 | -0.326 | -0.016* | -0.041 | -0.036 |
|  | (0.662) | (0.042) | (0.102) | (0.091) | (0.094) | (0.037) |  |  |
| Full-time non-agricultural work | -0.351 | -0.619 | -0.734 | -0.568 | -0.380 | -0.017* | -0.046 | -0.035 |
|  | (0.338) | (0.076) | (0.084) | (0.061) | (0.062) | (0.044) |  |  |
| Work status of mother (Ref. None/limited) |  |  |  |  |  |  |  |  |
| Full-time farming work (Mother) | 0.350* | 0.327 | 0.166 | 0.281* | 0.191* | 0.004 | 0.023 | 0.009 |
|  | (0.033) | (0.056) | (0.383) | (0.036) | (0.034) | (0.293) |  |  |
| Full-time non-agricultural work | -0.479 | -0.244 | -0.616 | -0.446 | -0.300 | -0.011 | -0.022 | -0.014 |
|  | (0.226) | (0.458) | (0.102) | (0.129) | (0.131) | (0.128) |  |  |
| Architecture type (Ref. concrete structure) |  |  |  |  |  |  |  |  |
| Adobe house | 0.377 | 0.705** | 0.595* | 0.559** | 0.375** | 0.015** | 0.054 | 0.037 |
|  | (0.066) | (0.003) | (0.015) | (0.002) | (0.002) | (0.007) |  |  |
| Wood/thatched house | 0.428 | 1.042*** | 0.769** | 0.746*** | 0.501*** | 0.020*** | 0.056 | 0.039 |
|  | (0.134) | (<0.001) | (0.005) | (<0.001) | (<0.001) | (<0.001) |  |  |
| Cave/Mongolian yurt/boat house/others | 0.356 | 0.387 | 0.223 | 0.322 | 0.218 | 0.006 | 0.018 | 0.008 |
|  | (0.209) | (0.282) | (0.475) | (0.196) | (0.193) | (0.433) |  |  |
| Neighborhood safety (Ref. very safe) |  |  |  |  |  |  |  |  |
| Somewhat safe | -0.064 | -0.197 | -0.038 | -0.100 | -0.068 | -0.002 | -0.010 | -0.005 |
|  | (0.613) | (0.108) | (0.794) | (0.294) | (0.290) | (0.532) |  |  |
| Not very safe | -0.223 | -0.162 | -0.035 | -0.140 | -0.096 | -0.001 | -0.006 | -0.001 |
|  | (0.459) | (0.595) | (0.898) | (0.555) | (0.550) | (0.867) |  |  |
| Not safe at all | 1.278** | 0.439 | 1.062* | 0.926** | 0.626** | 0.018 | 0.026 | 0.013 |
|  | (0.002) | (0.312) | (0.020) | (0.005) | (0.005) | (0.065) |  |  |
| Neighborhood cohesion (Ref. very close) |  |  |  |  |  |  |  |  |
| Somewhat close-knit | 0.409** | 0.077 | 0.049 | 0.179 | 0.123 | -0.000 | 0.018 | -0.000 |
|  | (0.003) | (0.576) | (0.747) | (0.109) | (0.100) | (0.992) |  |  |
| Not very close-knit | 0.487 | 0.195 | 0.479 | 0.387 | 0.261 | 0.008 | 0.014 | 0.008 |
|  | (0.205) | (0.600) | (0.246) | (0.188) | (0.186) | (0.351) |  |  |
| Not close-knit at all | 2.095** | 1.834* | 2.095** | 2.008*** | 1.354*** | 0.044** | 0.032 | 0.018 |
|  | (0.005) | (0.017) | (0.003) | (<0.001) | (<0.001) | (0.004) |  |  |
| Friendship (Ref. often) |  |  |  |  |  |  |  |  |
| Sometimes | 0.205 | 0.443* | 0.361 | 0.337* | 0.226* | 0.009* | 0.023 | 0.016 |
|  | (0.258) | (0.013) | (0.059) | (0.018) | (0.019) | (0.025) |  |  |
| Not very often | 0.185 | 0.810*** | 0.498* | 0.498** | 0.333** | 0.014** | 0.028 | 0.021 |
|  | (0.435) | (<0.001) | (0.035) | (0.005) | (0.005) | (0.003) |  |  |
| Never | 0.823*** | 1.282*** | 1.231*** | 1.112*** | 0.747*** | 0.029*** | 0.072 | 0.049 |
|  | (<0.001) | (<0.001) | (<0.001) | (<0.001) | (<0.001) | (<0.001) |  |  |
| Relationship with mother (Ref. Fair/Poor) |  |  |  |  |  |  |  |  |
| Good (Mother) | 0.058 | -0.201 | -0.495* | -0.213 | -0.139 | -0.011* | -0.016 | -0.021 |
|  | (0.795) | (0.341) | (0.022) | (0.207) | (0.221) | (0.015) |  |  |
| Relationship with father (Ref. Fair/Poor) |  |  |  |  |  |  |  |  |
| Good (Father) | -0.220 | -0.132 | 0.417* | 0.022 | 0.010 | 0.008 | 0.001 | 0.015 |
|  | (0.301) | (0.548) | (0.040) | (0.894) | (0.928) | (0.085) |  |  |
| Relative Health Status (Ref. Healthier) |  |  |  |  |  |  |  |  |
| About average | 0.321* | 0.094 | 0.522*** | 0.313** | 0.209** | 0.009** | 0.031 | 0.023 |
|  | (0.040) | (0.442) | (<0.001) | (0.003) | (0.003) | (0.001) |  |  |
| Less healthy | 0.195 | 0.331 | 0.098 | 0.208 | 0.141 | 0.004 | 0.013 | 0.006 |
|  | (0.430) | (0.112) | (0.687) | (0.287) | (0.284) | (0.473) |  |  |
| Confined to bed (Ref. No) |  |  |  |  |  |  |  |  |
| Yes | -0.335 | -0.017 | -0.000 | -0.117 | -0.082 | 0.001 | -0.005 | 0.001 |
|  | (0.341) | (0.958) | (1.000) | (0.656) | (0.647) | (0.860) |  |  |
| Hospitalized (Ref. No) |  |  |  |  |  |  |  |  |
| Yes | 0.310 | 0.377 | 0.516 | 0.401 | 0.269 | 0.011 | 0.011 | 0.008 |
|  | (0.527) | (0.418) | (0.322) | (0.326) | (0.328) | (0.312) |  |  |
| Ever receive vaccinations (Ref. No) |  |  |  |  |  |  |  |  |
| Yes | -0.118 | -0.335* | -0.670*** | -0.374* | -0.248* | -0.014*** | -0.025 | -0.025 |
|  | (0.535) | (0.050) | (<0.001) | (0.010) | (0.011) | (<0.001) |  |  |
| Not enough food during 0-5 (Ref. No) |  |  |  |  |  |  |  |  |
| Yes | 0.161 | 0.151 | 0.463** | 0.258* | 0.172* | 0.009** | 0.024 | 0.022 |
|  | (0.191) | (0.257) | (0.006) | (0.021) | (0.022) | (0.009) |  |  |
|  |  |  |  |  |  |  |  |  |
| Observations | 6,700 | 6,700 | 6,700 | 6,700 | 6,700 | 6,700 | 6,700 | 6,700 |
| R-squared | 0.275 | 0.331 | 0.342 | 0.428 | 0.558 | 0.773 |  |  |
| Covariates | YES | YES | YES | YES | YES | YES | YES | YES |

*Notes:* Standard errors were clustered at community level. Covariates were controlled in all six models, including age, gender, education, hukou status (rural/urban), marital status, log income and number of chronic diseases. LMM = linear mixed-effect model. Columns 1-3 show the estimates of the model using reversed cognitive test scores from each of the three waves, respectively. Column 4 shows the regression results of the model using the average reversed test scores (i.e., Pooled Avg). Columns 5, 6 replicate the regression results of the model using the level and rate estimated from LMM (i.e., Columns 7, 8 in Table 3). The standardized effect size estimates in Column 7 and Column 8 respectively represent the effect of a one SD change in childhood circumstances on the level and rate of cognitive aging (SDs of level and rate). P-values are shown in parentheses. Statistical significance: *** *p*<0.001, ** *p*<0.01, * *p*<0.05

**Table A3.** Comparing the exposure estimates of regressions with and without adjusting for education.

|  | (1) | (2) | (3) | (4) |
| --- | --- | --- | --- | --- |
|  | Level | Rate | Level | Rate |
| Education of father (Ref. Illiterate) |  |  |  |  |
| Elementary school and below | -0.724*** | -0.028*** | -0.394*** | -0.014*** |
|  | (0.000) | (0.000) | (0.000) | (0.000) |
| Middle school and above | -0.514** | -0.017* | -0.340* | -0.011* |
|  | (0.004) | (0.022) | (0.015) | (0.048) |
| Education of mother (Ref. Illiterate) |  |  |  |  |
| Elementary school and below | -0.511*** | -0.018** | -0.241* | -0.009 |
|  | (0.000) | (0.001) | (0.042) | (0.065) |
| Middle school and above | -1.471* | -0.057** | -1.080* | -0.044** |
|  | (0.019) | (0.010) | (0.028) | (0.006) |
| Work status of father (Ref. None/limited) |  |  |  |  |
| Full-time farming work (Farther) | -0.596* | -0.031** | -0.358 | -0.021* |
|  | (0.024) | (0.005) | (0.093) | (0.013) |
| Full-time non-agricultural work | -0.919*** | -0.040*** | -0.356 | -0.018* |
|  | (0.001) | (0.000) | (0.107) | (0.041) |
| Work status of mother (Ref. None/limited) |  |  |  |  |
| Full-time farming work (Mother) | 0.331** | 0.009 | 0.217* | 0.004 |
|  | (0.004) | (0.066) | (0.025) | (0.317) |
| Full-time non-agricultural work | -0.251 | -0.011 | -0.344 | -0.014 |
|  | (0.421) | (0.334) | (0.120) | (0.089) |
| Architecture type (Ref. concrete structure) |  |  |  |  |
| Adobe house | 0.440* | 0.021** | 0.309* | 0.015** |
|  | (0.013) | (0.006) | (0.022) | (0.008) |
| Wood/thatched house | 0.939*** | 0.041*** | 0.495** | 0.021*** |
|  | (0.000) | (0.000) | (0.001) | (0.001) |
| Cave/Mongolian yurt/boat house/others | 0.267 | 0.008 | 0.125 | 0.004 |
|  | (0.203) | (0.378) | (0.489) | (0.574) |
| Neighborhood safety (Ref. very safe) |  |  |  |  |
| Somewhat safe | -0.142 | -0.007 | -0.039 | -0.002 |
|  | (0.126) | (0.095) | (0.597) | (0.555) |
| Not very safe | -0.040 | 0.000 | -0.089 | -0.001 |
|  | (0.824) | (0.971) | (0.590) | (0.821) |
| Not safe at all | 0.828** | 0.023 | 0.674** | 0.017 |
|  | (0.004) | (0.070) | (0.007) | (0.136) |
| Neighborhood cohesion (Ref. very close) |  |  |  |  |
| Somewhat close-knit | 0.035 | -0.003 | 0.034 | -0.002 |
|  | (0.705) | (0.449) | (0.662) | (0.500) |
| Not very close-knit | 0.350 | 0.009 | 0.289 | 0.007 |
|  | (0.163) | (0.437) | (0.181) | (0.398) |
| Not close-knit at all | 1.617** | 0.061** | 1.308** | 0.042* |
|  | (0.001) | (0.007) | (0.002) | (0.012) |
| Friendship (Ref. often) |  |  |  |  |
| Sometimes | 0.277* | 0.010 | 0.252** | 0.010* |
|  | (0.026) | (0.074) | (0.010) | (0.018) |
| Not very often | 0.681*** | 0.029*** | 0.270* | 0.013** |
|  | (0.000) | (0.000) | (0.040) | (0.010) |
| Never | 1.366*** | 0.059*** | 0.795*** | 0.031*** |
|  | (0.000) | (0.000) | (0.000) | (0.000) |
| Relationship with mother (Ref. Fair/Poor) |  |  |  |  |
| Good (Mother) | -0.276 | -0.019** | -0.131 | -0.013** |
|  | (0.059) | (0.002) | (0.289) | (0.006) |
| Relationship with father (Ref. Fair/Poor) |  |  |  |  |
| Good (Father) | -0.234 | 0.000 | -0.048 | 0.007 |
|  | (0.072) | (0.994) | (0.679) | (0.105) |
| Relative Health Status (Ref. Healthier) |  |  |  |  |
| About average | 0.321*** | 0.014*** | 0.203** | 0.010** |
|  | (0.000) | (0.000) | (0.004) | (0.001) |
| Less healthy | 0.168 | 0.006 | 0.155 | 0.005 |
|  | (0.463) | (0.487) | (0.268) | (0.356) |
| Confined to bed (Ref. No) |  |  |  |  |
| Yes | 0.231 | 0.011 | -0.034 | 0.003 |
|  | (0.338) | (0.237) | (0.859) | (0.681) |
| Hospitalized (Ref. No) |  |  |  |  |
| Yes | 0.386 | 0.027 | 0.258 | 0.015 |
|  | (0.267) | (0.057) | (0.409) | (0.235) |
| Ever receive vaccinations (Ref. No) |  |  |  |  |
| Yes | -0.725*** | -0.035*** | -0.303** | -0.016*** |
|  | (0.000) | (0.000) | (0.004) | (0.000) |
| Not enough food during 0-5 (Ref. No) |  |  |  |  |
| Yes | 0.264** | 0.013*** | 0.155* | 0.008* |
|  | (0.003) | (0.001) | (0.036) | (0.011) |
|  |  |  |  |  |
| Observations | 6,042 | 6,042 | 6,042 | 6,042 |
| R-squared | 0.367 | 0.655 | 0.551 | 0.769 |
|  |  |  |  |  |
| Other covariates in the full model except education | YES | YES | YES | YES |
| Education | NO | NO | YES | YES |

*Notes:* Standard errors were clustered at community level. Column 1 and 3 show the regression results using the level of cognitive deficit as outcomes, whereas column 2 and 4 show the result using the rate of cognitive decline as outcomes. The models for column 1-2 only adjusted for baseline covariates except education, including age, gender, hukou status (rural/urban), marital status, log income and number of chronic diseases, while the models for column 3-4 additionally controlled for education. Regressions were weighted at individual level with household and individual non-response adjustment. P-values are shown in parentheses. Statistical significance: *** *p*<0.001, ** *p*<0.01, * *p*<0.05

**Table A4.** Comparing the exposure estimates of regressions cumulatively adjusted for later-life family wealth, health and health behaviors, and social engagements.

|  | (1) | (2) | (3) | (4) | (5) | (6) |
| --- | --- | --- | --- | --- | --- | --- |
|  | Level | Rate | Level | Rate | Level | Rate |
| Education of father (Ref. Illiterate) |  |  |  |  |  |  |
| Elementary school and below | -0.388*** | -0.014*** | -0.387*** | -0.014*** | -0.369*** | -0.013*** |
|  | (<0.001) | (<0.001) | (<0.001) | (<0.001) | (<0.001) | (<0.001) |
| Middle school and above | -0.335* | -0.011 | -0.324* | -0.010 | -0.358** | -0.011* |
|  | (0.016) | (0.051) | (0.020) | (0.063) | (0.009) | (0.045) |
| Education of mother (Ref. Illiterate) |  |  |  |  |  |  |
| Elementary school and below | -0.235 | -0.008 | -0.235* | -0.009 | -0.195 | -0.008 |
|  | (0.053) | (0.074) | (0.049) | (0.068) | (0.100) | (0.106) |
| Middle school and above | -0.936* | -0.041** | -0.948* | -0.042** | -0.824* | -0.039** |
|  | (0.029) | (0.006) | (0.024) | (0.004) | (0.047) | (0.007) |
| Work status of father (Ref. None/limited) |  |  |  |  |  |  |
| Full-time farming work (Farther) | -0.345 | -0.021* | -0.345 | -0.021* | -0.338 | -0.020* |
|  | (0.108) | (0.015) | (0.102) | (0.014) | (0.103) | (0.014) |
| Full-time non-agricultural work | -0.337 | -0.018* | -0.325 | -0.017 | -0.296 | -0.016 |
|  | (0.128) | (0.046) | (0.136) | (0.051) | (0.166) | (0.064) |
| Work status of mother (Ref. None/limited) |  |  |  |  |  |  |
| Full-time farming work (Mother) | 0.214* | 0.004 | 0.215* | 0.004 | 0.206* | 0.004 |
|  | (0.027) | (0.326) | (0.024) | (0.325) | (0.027) | (0.325) |
| Full-time non-agricultural work | -0.211 | -0.011 | -0.230 | -0.012 | -0.215 | -0.011 |
|  | (0.262) | (0.169) | (0.229) | (0.145) | (0.253) | (0.177) |
| Architecture type (Ref. concrete structure) |  |  |  |  |  |  |
| Adobe house | 0.314* | 0.015** | 0.313* | 0.016** | 0.293* | 0.015** |
|  | (0.015) | (0.007) | (0.015) | (0.006) | (0.024) | (0.008) |
| Wood/thatched house | 0.497*** | 0.021*** | 0.487** | 0.021*** | 0.458** | 0.020** |
|  | (<0.001) | (<0.001) | (0.001) | (<0.001) | (0.002) | (0.001) |
| Cave/Mongolian yurt/boat house/others | 0.106 | 0.004 | 0.106 | 0.004 | 0.083 | 0.004 |
|  | (0.558) | (0.617) | (0.558) | (0.577) | (0.644) | (0.629) |
| Neighborhood safety (Ref. very safe) |  |  |  |  |  |  |
| Somewhat safe | -0.057 | -0.002 | -0.054 | -0.002 | -0.045 | -0.002 |
|  | (0.424) | (0.467) | (0.453) | (0.502) | (0.527) | (0.587) |
| Not very safe | -0.113 | -0.002 | -0.109 | -0.002 | -0.070 | -0.000 |
|  | (0.493) | (0.749) | (0.509) | (0.769) | (0.665) | (0.944) |
| Not safe at all | 0.648** | 0.016 | 0.643** | 0.016 | 0.601* | 0.015 |
|  | (0.009) | (0.151) | (0.010) | (0.161) | (0.014) | (0.185) |
| Neighborhood cohesion (Ref. very close) |  |  |  |  |  |  |
| Somewhat close-knit | 0.036 | -0.002 | 0.023 | -0.003 | 0.004 | -0.004 |
|  | (0.634) | (0.514) | (0.760) | (0.413) | (0.954) | (0.291) |
| Not very close-knit | 0.283 | 0.007 | 0.277 | 0.007 | 0.223 | 0.006 |
|  | (0.189) | (0.407) | (0.196) | (0.425) | (0.290) | (0.502) |
| Not close-knit at all | 1.317** | 0.042* | 1.285** | 0.041* | 1.215** | 0.039* |
|  | (0.002) | (0.012) | (0.002) | (0.014) | (0.002) | (0.017) |
| Friendship (Ref. often) |  |  |  |  |  |  |
| Sometimes | 0.254** | 0.010* | 0.259** | 0.011* | 0.235* | 0.010* |
|  | (0.010) | (0.018) | (0.008) | (0.016) | (0.016) | (0.024) |
| Not very often | 0.278* | 0.013** | 0.276* | 0.013** | 0.181 | 0.010* |
|  | (0.034) | (0.009) | (0.034) | (0.009) | (0.166) | (0.047) |
| Never | 0.791*** | 0.030*** | 0.784*** | 0.030*** | 0.667*** | 0.027*** |
|  | (<0.001) | (<0.001) | (<0.001) | (<0.001) | (<0.001) | (<0.001) |
| Relationship with mother (Ref. Fair/Poor) |  |  |  |  |  |  |
| Good (Mother) | -0.127 | -0.013** | -0.117 | -0.013** | -0.085 | -0.012* |
|  | (0.297) | (0.006) | (0.338) | (0.008) | (0.475) | (0.014) |
| Relationship with father (Ref. Fair/Poor) |  |  |  |  |  |  |
| Good (Father) | -0.055 | 0.007 | -0.054 | 0.007 | -0.091 | 0.006 |
|  | (0.634) | (0.112) | (0.641) | (0.104) | (0.428) | (0.177) |
| Relative Health Status (Ref. Healthier) |  |  |  |  |  |  |
| About average | 0.192** | 0.009** | 0.193** | 0.010** | 0.170* | 0.009** |
|  | (0.007) | (0.002) | (0.008) | (0.002) | (0.017) | (0.004) |
| Less healthy | 0.151 | 0.005 | 0.144 | 0.005 | 0.123 | 0.004 |
|  | (0.259) | (0.355) | (0.282) | (0.370) | (0.374) | (0.435) |
| Confined to bed (Ref. No) |  |  |  |  |  |  |
| Yes | -0.058 | 0.002 | -0.074 | 0.002 | -0.075 | 0.001 |
|  | (0.763) | (0.741) | (0.699) | (0.826) | (0.685) | (0.867) |
| Hospitalized (Ref. No) |  |  |  |  |  |  |
| Yes | 0.237 | 0.014 | 0.246 | 0.015 | 0.214 | 0.014 |
|  | (0.448) | (0.252) | (0.432) | (0.227) | (0.491) | (0.251) |
| Ever receive vaccinations (Ref. No) |  |  |  |  |  |  |
| Yes | -0.292** | -0.016*** | -0.280** | -0.015*** | -0.238* | -0.014*** |
|  | (0.005) | (<0.001) | (0.007) | (<0.001) | (0.016) | (<0.001) |
| Not enough food during 0-5 (Ref. No) |  |  |  |  |  |  |
| Yes | 0.157* | 0.008* | 0.153* | 0.008* | 0.148* | 0.008* |
|  | (0.036) | (0.012) | (0.041) | (0.013) | (0.042) | (0.012) |
|  |  |  |  |  |  |  |
| Observations | 6,042 | 6,042 | 6,042 | 6,042 | 6,016 | 6,016 |
| R-squared | 0.553 | 0.769 | 0.554 | 0.770 | 0.563 | 0.773 |
|  |  |  |  |  |  |  |
| Covariates in the full model (including education) | YES | YES | YES | YES | YES | YES |
| Later-life family wealth | YES | YES | YES | YES | YES | YES |
| Later-life health and health behaviors | NO | NO | YES | YES | YES | YES |
| Later-life social engagements | NO | NO | NO | NO | YES | YES |

*Notes:* Standard errors were clustered at community level. Other than the covariates controlled in the full model, including age, gender, education, hukou status (rural/urban), marital status, log income and number of chronic diseases, the models for columns 1-6 also cumulatively controlled for three other sets of covariates, including later-life family wealth, health and health behaviors, and social engagements. Later-life family wealth represents the total non-housing financial wealth of the respondent and spouse at the baseline. Later-life health captures the health risks related to dementia, including hypertension, heart diseases, diabetes, and dyslipidemia at the baseline; and health behaviors measure whether the respondents were still smoking or drinking at the baseline. Later-life social engagements measure whether the respondents participated in any of the following social activities in the last month: “Interacted with friend”, “Played Ma-jong, chess, cards, or went to a community club”, “Went to a sporting event, participated in a social group, or participated in some other sort of club”, “Took part in a community-related organization”, “Took part in voluntary or charity work”, “Attended an educational or training course”, where we included the measures collected from Wave 1 to Wave 3 to capture the respondents’ long-term patterns of social activities. Regressions were weighted at individual level with household and individual non-response adjustment. P-values are shown in parentheses. Statistical significance: *** *p*<0.001, ** *p*<0.01, * *p*<0.05

**Figure A1.** The distributions of cognitive deficit of sample collected in three waves.


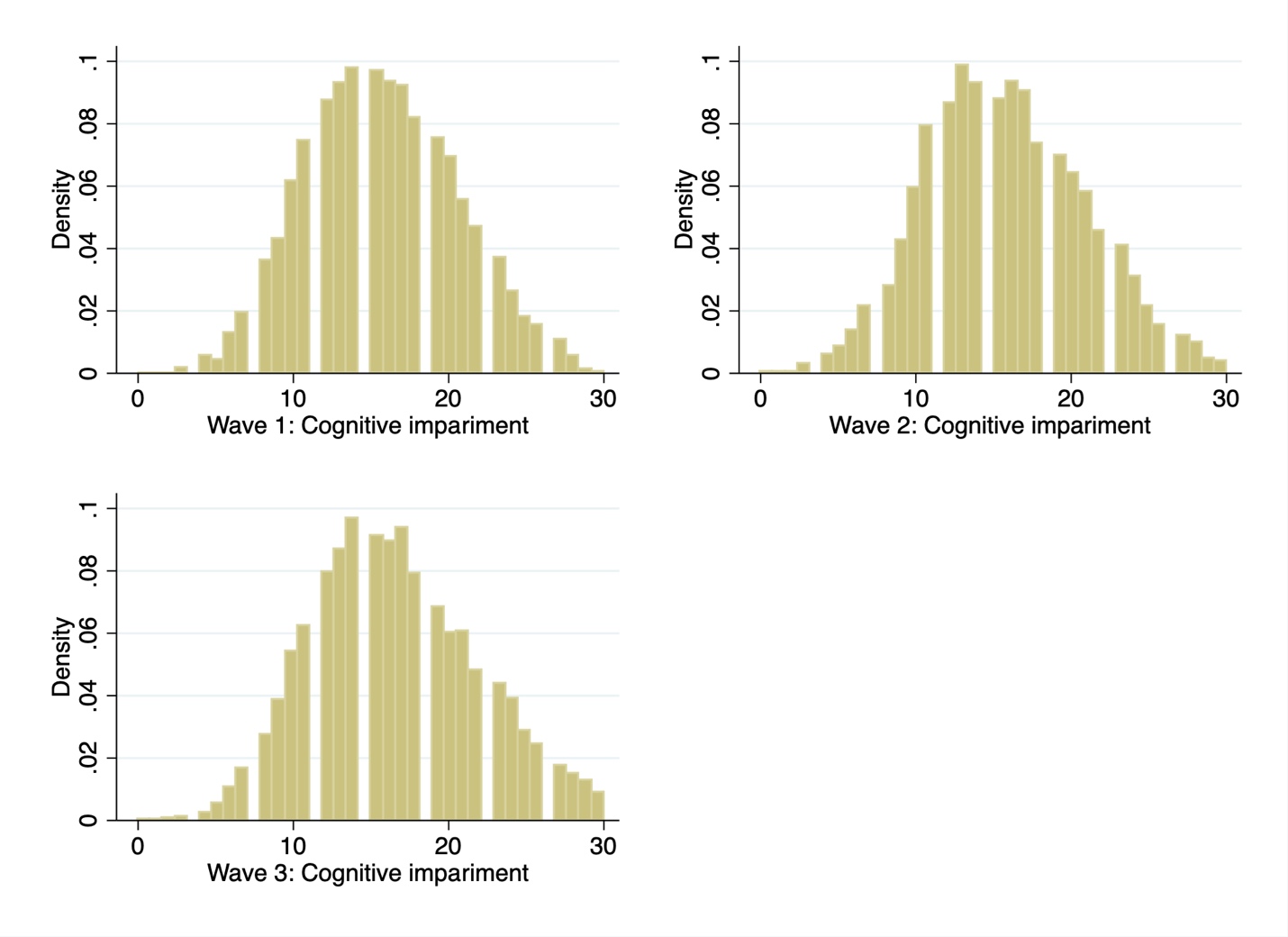


*Note*: Each histogram shows the distribution of cognitive deficit for each measured wave among the sample used in linear regression (N=6,700)

**Figure A2.** A flow chart of consecutive sample selection and data analysis


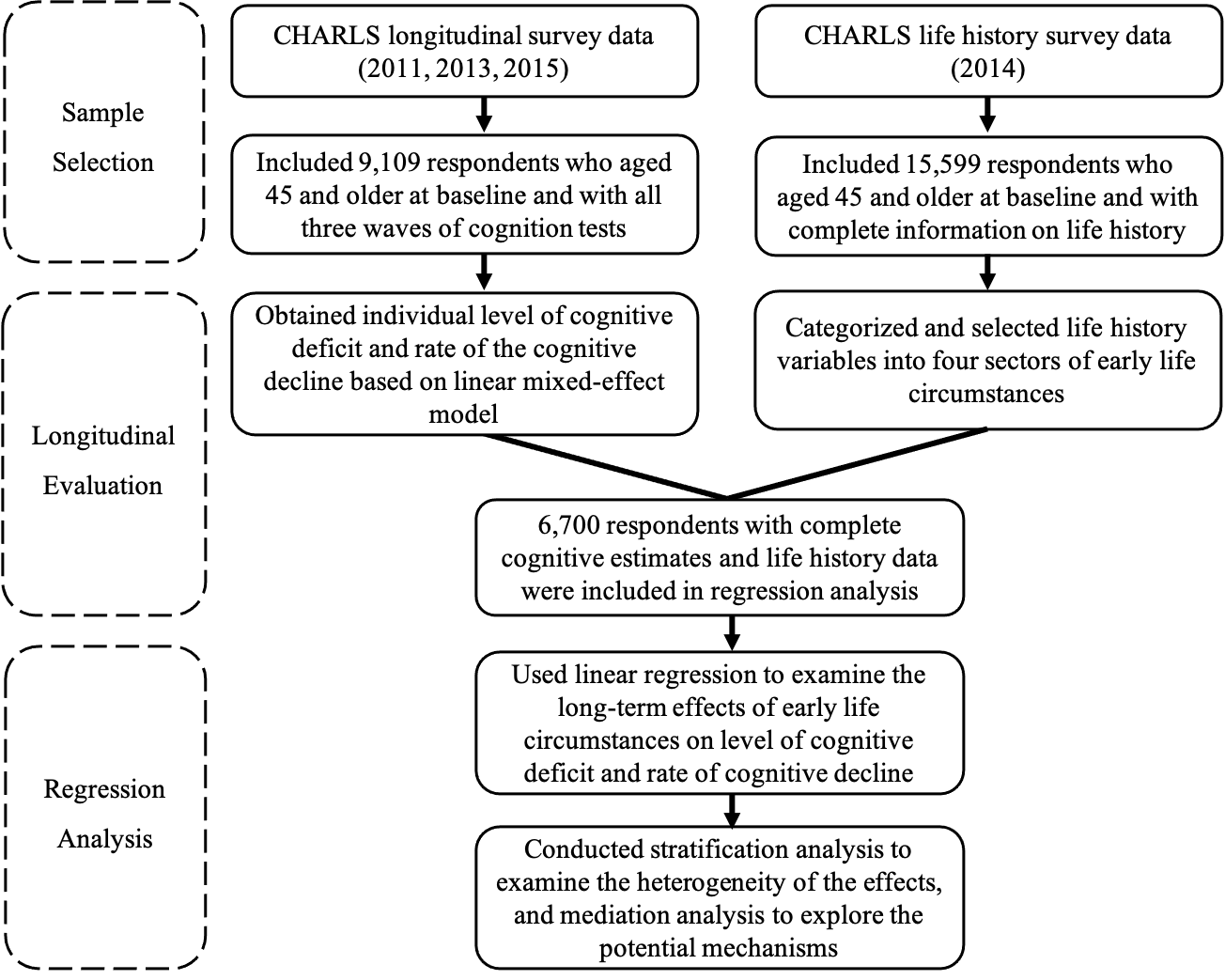


**Figure A3.** Estimated rate of cognitive decline and baseline level of cognitive deficit


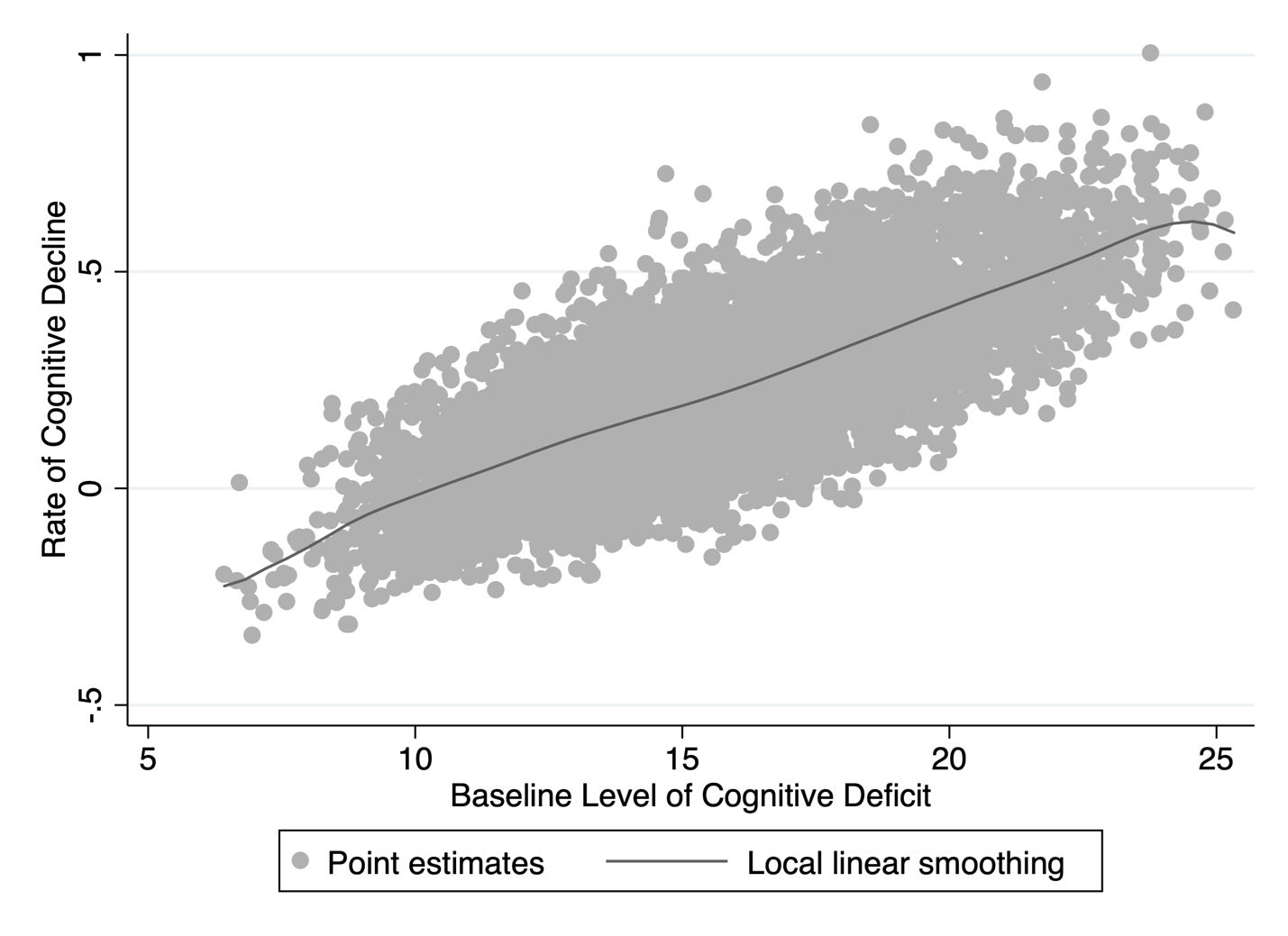


*Notes:* The relationship between rate of cognitive decline and baseline level of cognitive deficit is illustrated above. Plotted points represent the individual estimates of level and rate. The regression line was fitted by local linear smoothing (N=6,700). Pearson’s correlation coefficient=0.751, p<0.001.

**Figure A4.** The effects of childhood circumstances on cognitive aging among male and female

Panel A. Childhood circumstances and level of cognitive deficit by gender


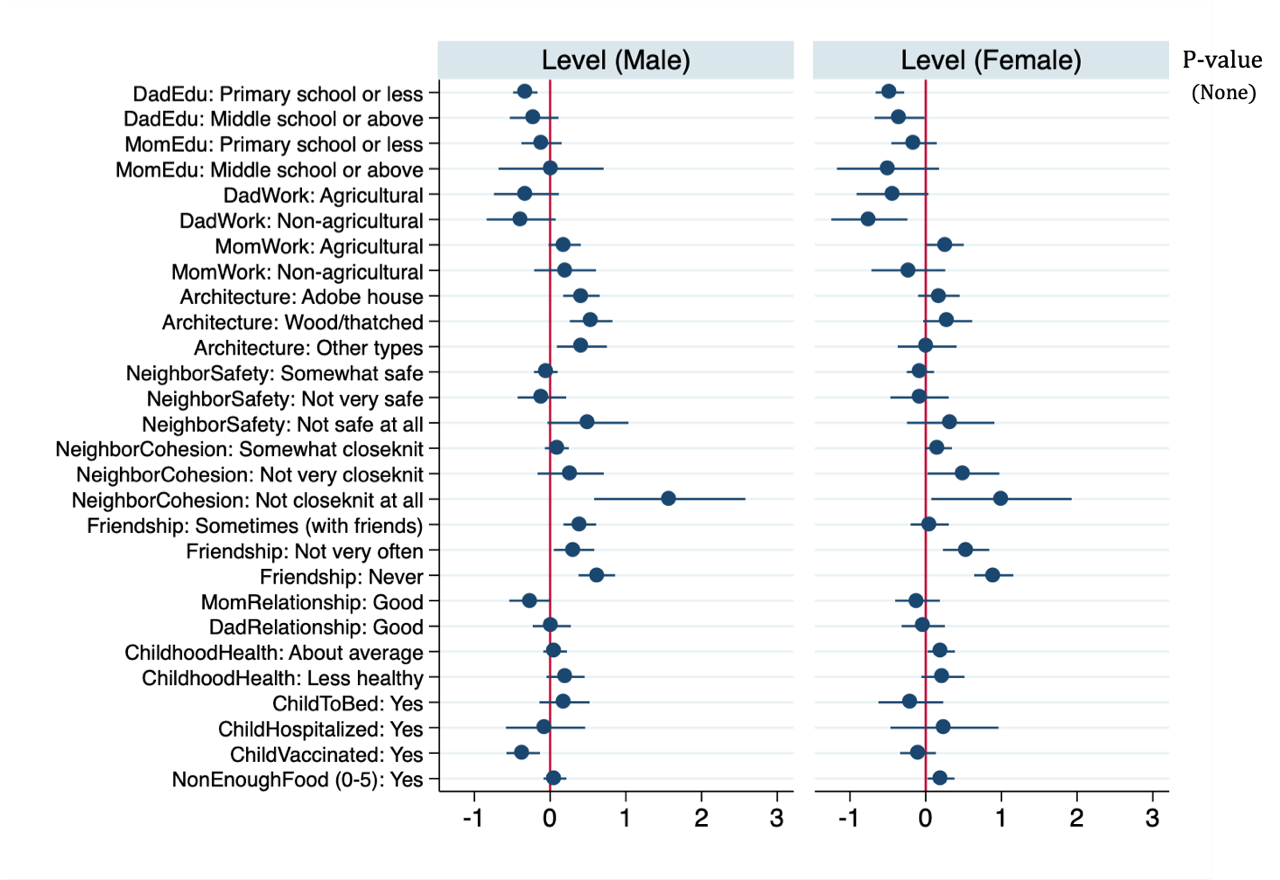


Panel B. Childhood circumstances and rate of cognitive decline by gender

**
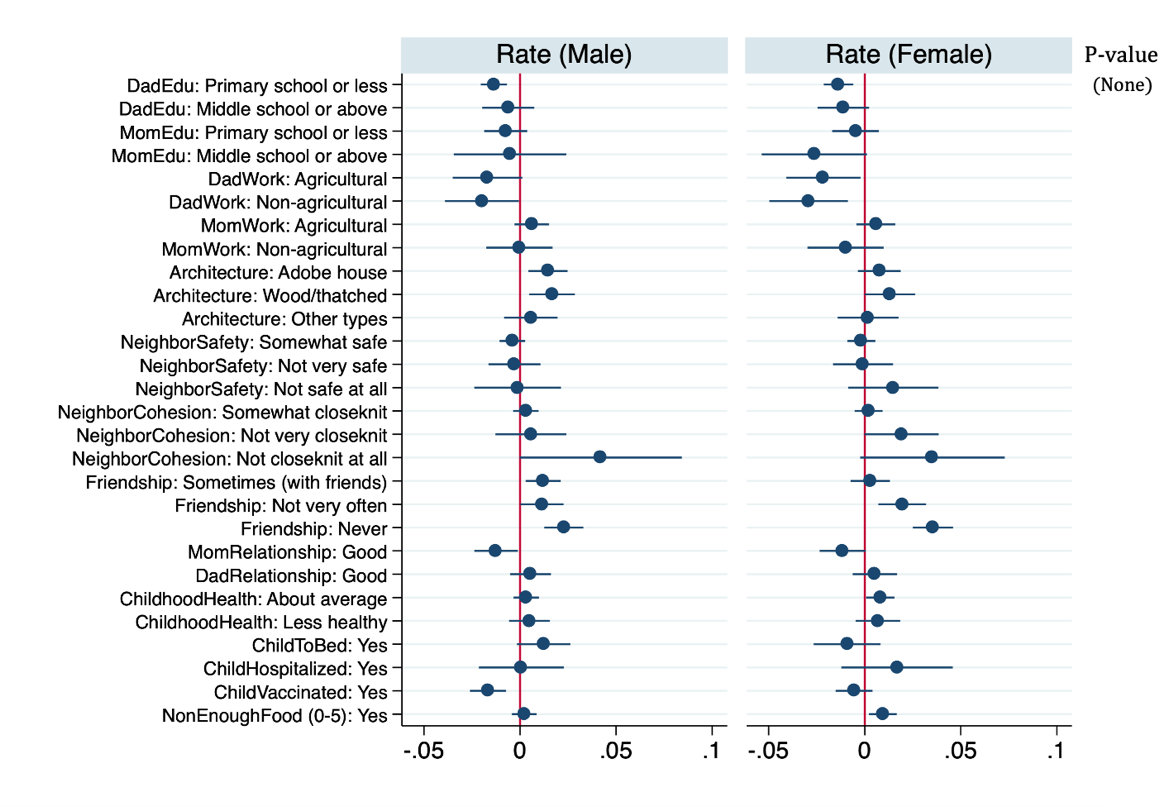
**

*Notes:* Coefficient plots of the childhood circumstances on level of cognitive deficit (Panel A) and rate of decline (Panel B) among male and female. The cross-equation test was conducted respectively to examine the statistical difference between the coefficients in two linear regressions. P-value was calculated based on Chow/Wald test, showing at the rightmost side of each panel (Only significant results are illustrated, whereas other estimates are available upon request. Statistical significance: *** p<0.001, ** p<0.01, * p<0.05).**Figure A5.** The effects of childhood circumstances on cognitive aging among people with rural and urban hukou

Panel A. Childhood circumstances and level of cognitive deficit by rural/urban hukou


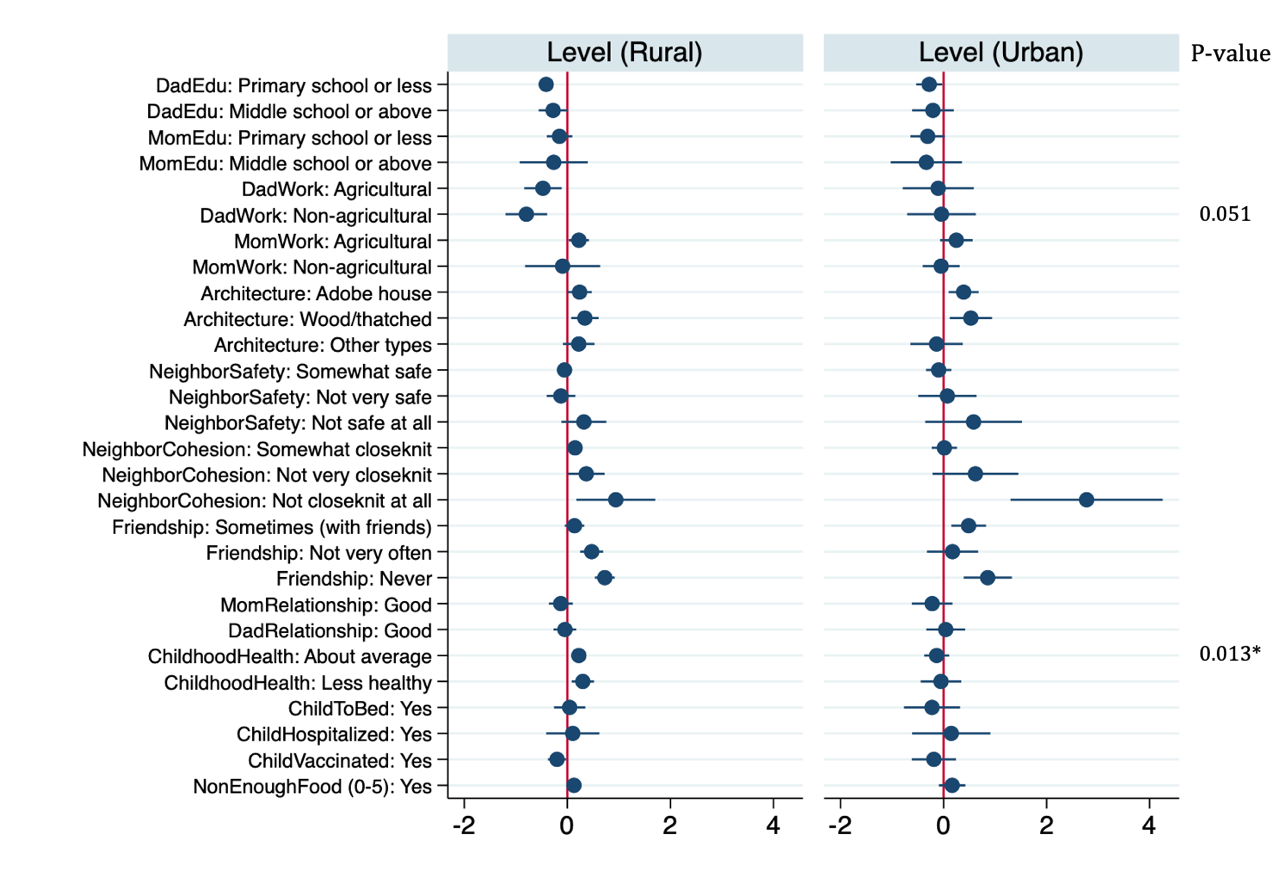


Panel B. Childhood circumstances and rate of cognitive decline by rural/urban hukou

**
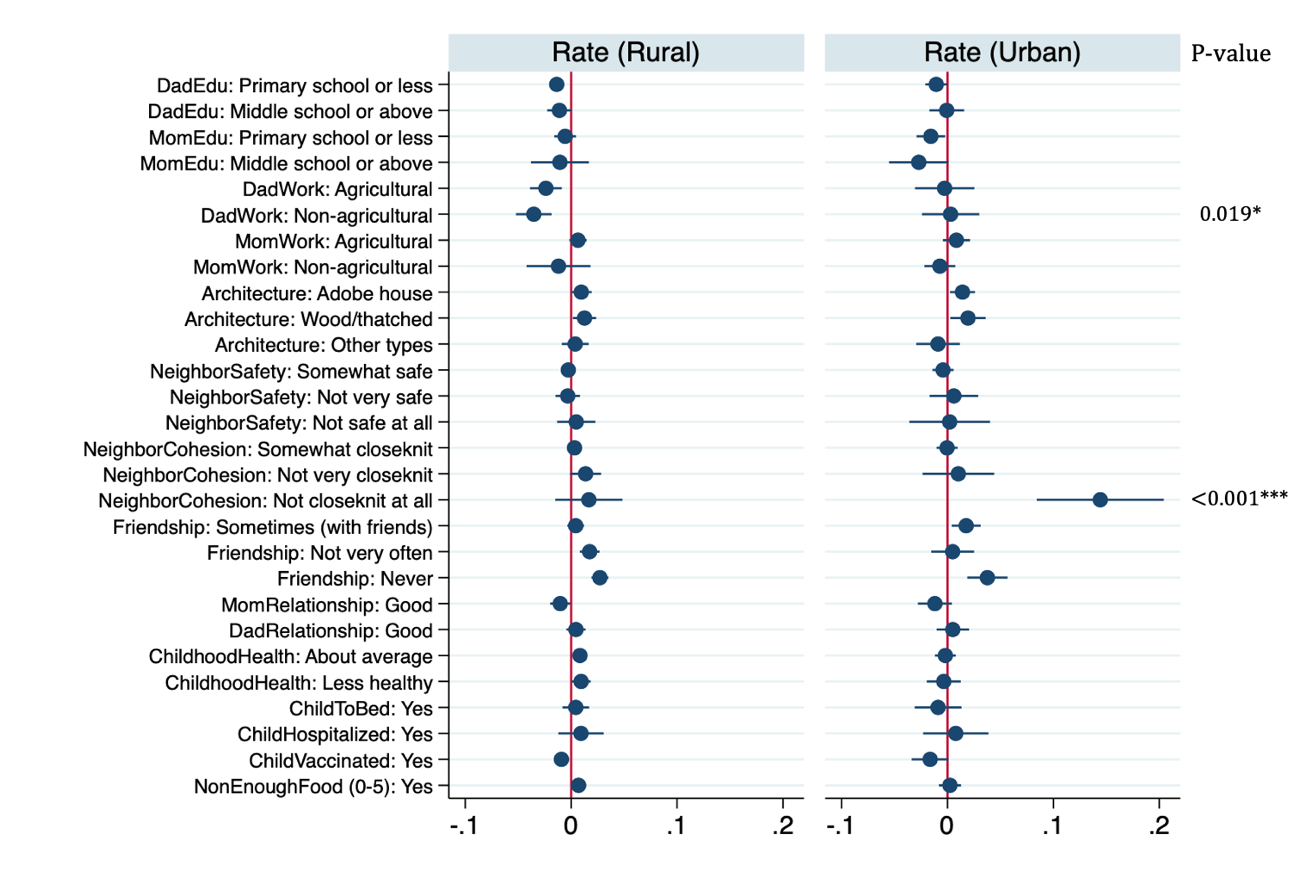
**

*Notes:* Coefficient plots of the childhood circumstances on level of cognitive deficit (Panel A) and rate of decline (Panel B) among individuals with rural hukou status at the baseline compared to those with urban hukou status. Hukou status (rural/urban) is a special identifier for the population in China as every Chinese citizen should legally register as either agricultural or non-agricultural residency status (normally referred to as rural vs. urban). Detailed definition and implications of rural/urban hukou status can be found in Appendix Table C2. The cross-equation test was conducted respectively to examine the statistical difference between the coefficients in two linear regressions. P-value was calculated based on Chow/Wald test, showing at the rightmost side of each panel. (Only significant/marginally significant results are illustrated, whereas other estimates are available upon request. Statistical significance: *** p<0.001, ** p<0.01, * p<0.05).

**Appendix B. Data**

**Data Sampling**

The CHARLS national baseline survey (i.e., wave 1, 2011-2012) adopted a stratified, multi-stage (county/district - village/community - household), probability proportional to population (PPS) random sampling strategy. In specific, 150 counties or urban districts were randomly chosen with probability proportional to population (PPS) out of all county-level units from all provinces except Tibet, where the county units were stratified by region, urban-rural, and by GDP per capita (Zhao et al., 2016, 2014). Then for each county, three administrative villages/urban neighborhoods (i.e., the primary sampling units, PSUs) were randomly selected with PPS sampling, which resulted in a total of 450 villages/neighborhoods. The sampling process, hence, makes CHARLS national representative and representative of both rural and urban areas in China, with the selected counties and districts representing 28 provinces out of 30 (Zhao et al., 2014). To obtain the most dated household listings, CHARLS developed a mapping/listing software (CHARLS-GIS) to list all dwellings units within a building based on Google-earth map images, to create sampling frames. From the frames, approximately 20 age eligible households were sampled per PSU; and in each sampled household, one person aged 45 or above was randomly selected as the main respondent, and his/her spouse was also interviewed (Zhao et al., 2020, 2016). Finally, a total of 17,708 individuals in 10,257 households were included in the national baseline survey. The respondents were followed up every two years since wave 1 (2011-2012), and a refreshment sample of 45-46-years-olds and his/her spouse were randomly drawn from each sampled household in wave 2 (2013), and in wave 3 (2015) to fully ensure the sample representativeness of the 45+ population (as the baseline respondents got older in later waves) (Zhao et al., 2020). In addition to the biennial core survey waves (2011, 2013. 2015), a life history survey was conducted in 2014 to collect detailed information on respondents’ life course circumstances.

**Data Collection and Administration**

As for data collection, the cognitive assessments were longitudinally taken in 2011 and 2013 and 2015, and the childhood circumstances of respondents was systematically collected in the 2014 survey. The surveys were mainly administered by Peking University. All survey workers were trained by CHARLS staff members with a standard protocol, and two interviewers were sent to each county-level unit to interview about 72 households and required to conduct the face-to-face interviews in respondents’ homes. Data were collected using a computer-assisted personal interview (CAPI) system, and a quality assurance program was also carried out to ensure the reliability of the survey (Zhao et al., 2020, 2016).

**Ethical Approval and Informed Consent**

The study protocols for all the CHARLS waves obtained ethical approval from the Institutional Review Board (IRB) at Peking University. Each respondent who agreed to participate in the survey was asked to sign two copies of the informed consent, and the written informed consent was obtained from all study participants, and was electronically scanned and archived (Zhao et al., 2020).

**Appendix C. Variables**

**Table C1.** The Construction and the Conceptualization of the Measures of Childhood Circumstances.

| Variables | Questions in the Survey | Construction of Variables | Conceptualization of Variables |
| --- | --- | --- | --- |
| Education of father | What is the highest level of education your biological father completed? (Categorical)  1. No formal education (illiterate); 2. Did not finish primary school but capable of reading or writing; 3. Sishu/Home School; 4. Graduate from elementary school; 5. Graduate from middle school; 6. Graduate from high school; 7. Graduate from vocational school; 8. Graduate from two/three Year College/Associate degree; 9. Graduate from Four Year College/Bachelor's degree; 10. Graduate from Post-graduate, Master degree; 11. Graduate from Post-graduate, PhD degree. | We re-categorized father’s education level into three groups:  1. Illiterate (=1);  2. Elementary school and below (=2-4);  3. Middle school and above (=5-11). | Parental education, as a measure of childhood socioeconomic status (SES). |
| Education of mother | What is the highest level of education your biological mother completed? (Categorical)  1. No formal education (illiterate); 2. Did not finish primary school but capable of reading or writing; 3. Sishu/Home School; 4. Graduate from elementary school; 5. Graduate from middle school; 6. Graduate from high school; 7. Graduate from vocational school; 8. Graduate from two/three Year College/Associate degree; 9. Graduate from Four Year College/Bachelor's degree; 10. Graduate from Post-graduate, Master degree; 11. Graduate from Post-graduate, PhD degree. | We re-categorized mother’s education level into three groups:  1. Illiterate (=1);  2. Elementary school and below (=2-4);  3. Middle school and above (=5-11). | Parental education, as a measure of childhood socioeconomic status (SES). |
| Work status of father | How much of your childhood before you were age 17 did your male guardian either work for pay or work in a family business?  1. All of my childhood;  2. Part of my childhood;  3. None of my childhood.  What was your male guardian’s usual occupation when you were growing up before you were 17? Please specify occupation in detail, including the department, key responsibility and the position.  1. Farming;  2. Non-agricultural. | Based on the two questions, we specified whether respondents’ father had full-time job or not during their childhood; and if so, what type of jobs their father did. This leaded to three categories:  1. None or limited work;  2. Full-time farming work;  3. Full-time non-agricultural work. | Parental occupation/work status, as a measure of childhood SES. |
| Work status of mother | How much of your childhood before you were age 17 did your female guardian either work for pay or work in a family business?  1. All of my childhood;  2. Part of my childhood;  3. None of my childhood.  What was your female guardian’s usual occupation when you were growing up before you were 17? Please specify occupation in detail, including the department, key responsibility and the position.  1. Farming;  2. Non-agricultural. | Based on the two questions, we specified whether respondents’ mother had full-time job or not during their childhood; and if so, what type of jobs their mother did. This leaded to three categories:  1. None or limited work;  2. Full-time farming work;  3. Full-time non-agricultural work. | Parental occupation/work status, as a measure of childhood SES. |
| Architecture type of first residence house | From your birth/Year, what is the architectural type of your first residence?  1. Concrete structure, Built with bricks and wood;  2. Adobe house;  3. Wood house/Thatched houses;  4. Cave;  5. Mongolian yurt;  6. Boat house;  7. Others; | We re-categorized the architectural type of first residence into four groups:  1. Concrete structure;  2. Adobe house;  3. Wood house/thatched houses;  4. Cave/Mongolian yurt/boat house/others.  The categorization was based on the economic value that the housing structure might reflect, where we classified “Cave”, “Mongolian yurt”, “Boat house” and “Others” into one group. | Housing characteristics, as a proxy to childhood family economic and financial status (i.e., SES) |
| Neighborhood safety | Was it safe being out alone at night in the neighborhood where you lived as a child? Is it very safe, somewhat safe, not very safe or not safe at all?  1. Very safe;  2. Somewhat safe;  3. Not very safe;  4. Note safe at all. | The categories were identical to the survey question. | Neighborhood safety, as a measure of childhood neighborhood social environment. |
| Neighborhood cohesion | Were the neighbors of the place where you lived as a child very close-knit? Is it very close-knit, somewhat close-knit, not very close-knit or not close-knit at all?  1. Very close-knit;  2. Somewhat close-knit;  3. Not very close-knit;  4. Not close-knit at all. | The categories were identical to the survey question. | Neighborhood cohesion, as a measure of childhood neighborhood social environment. |
| Friendship | When you were a child, did you often have a group of friends that you felt comfortable spending time with? Is it often, sometimes, not very often or never?  1. Often;  2. Sometimes;  3. Not very often;  4. Never. | The categories were identical to the survey question. | Friendship, as a measure of childhood social relationships. |
| Relationship with father | How would you rate your relationship with your male guardian when you were growing up?  1. Excellent  2. Very good  3. Good  4. Fair  5. Poor | We re-categorized the relationship with father into two groups:  1. Fair/Poor (=4 or 5);  2. Good (=1 or 2 or 3). | Relationships with parents, as a measure of childhood social relationships. |
| Relationship with mother | How would you rate your relationship with your female guardian when you were growing up?  1. Excellent  2. Very good  3. Good  4. Fair  5. Poor | We re-categorized the relationship with mother into two groups:  1. Fair/Poor (=4 or 5);  2. Good (=1 or 2 or 3). | Relationships with parents, as a measure of childhood social relationships. |
| Relative health status compared to peers | Before you were 15 years old (including 15 years old), would you say that compared to other children of the same age, you were  1. Much healthier  2. Somewhat healthier  3. About average  4. Somewhat less healthy  5. Much less healthy | We re-categorized the childhood health into three groups:  1. Healthier (=1 or 2);  2. About average (=3);  3. Less healthy (=4 or 5). | Childhood self-reported health status, as a measure of general childhood health conditions. |
| Ever confined to bed more than one month | Before you were 15 years old (including 15 years old), because of a health condition, were you ever confined to bed or home for a month or more?  1. Yes  2. No | The categories were identical to the survey question. We used “No” as the reference group. | Disadvantaged health situation, as an objective measure of childhood health conditions. |
| Ever hospitalized | Before you were 15 years old (including 15 years old), because of a health condition, were you ever hospitalized for a month or more?  1. Yes  2. No | The categories were identical to the survey question. We used “No” as the reference group. | Disadvantaged health situation, as an objective measure of childhood health conditions. |
| Ever receive any vaccinations (before 15 years old) | Before you were 15 years old (including 15 years old), have you received any vaccinations?  1. Yes  2. No | The categories were identical to the survey question. We used “No” as the reference group. | Preventive care, as a measure of childhood health resources. |
| Not enough food during 0-5 years old | When you were a child before age 17 was there ever a time when your family did not have enough food to eat?  1. Yes  2. No  At what age ranges did this (your family had no enough food to eat) happen? (Multiple answers are allowed)  1. Age 0-5  2. Age 6-12  3. Age 13-17 | Based on these two questions, we specified whether the respondent had enough food or not during age 0-5:  1. No;  2. Yes.  We used “No” as the reference group. | Childhood nutritional status; chose age 0-5 as it is a critical stage of brain development. |

*Notes*: The covariates were constructed based on the CHARLS life history survey, which was collected in 2014.

**Table C2.** The Construction and Definition of Covariates and Mediators

| Variables | Construction of Variables | Definition of Variables |
| --- | --- | --- |
| Age | Age denoted the respondent’s age in years at the baseline (i.e., wave 1), which was calculated by the respondent’s birth year and month minus the interview year and month. | Age in years at the baseline. |
| Gender | Gender was reported in each wave of CHARLS surveys. There were two categories: 1. Male; 2. Female. | Biological gender/sex, male and female. |
| Education level | The highest level of education that the respondent attained was self-reported in CHARLS in categories (e.g., primary school, middle school, college). Considering the high prevalence of lower education among the study sample, we categorized it into four groups: 1. Illiterate or informal education; 2. Primary school; 3. Middle school; High school and above. | Educational attainment (categorical). |
| Hukou status | Respondents were asked to report their hukou status, which is a population registration system used in China that indicate individual’s rural or urban residency status. Specifically, agricultural hukou was classified as rural hukou, while non-agricultural hukou and unified residence hukou were classified as urban hukou.  1. Urban  2. Rural | Hukou is a population registration system that has long been used in China. Every Chinese citizen is required to legally register in the system, as either agricultural or non-agricultural residency (normally referred to as rural vs. urban).  Hukou status (rural/urban) often determines the social programs (and benefits) that individuals are eligible for, and affects many aspects of life such as school enrollment, real estate transaction, public insurance coverage and health services. It thus encompasses rich information on individuals’ social status and welfare benefits.  Therefore, we used hukou as the measure of respondents’ rural or urban status. |
| Marital Status | In CHARLS, respondents were asked to indicate their marital status (e.g., married, divorced, widowed). We categorized it into two categories: 1. Married with spouse present; 2. Not. | Baseline marital status (i.e., married with spouse present or not). |
| Log annual per capita income | It represented log annual per capita income in the household, which was calculated using all the sources of incomes reported in the past year. | Baseline annual income (in the past year) |
| The number of chronic diseases | We added up the chronic diseases that individuals self-reported at the baseline, including hypertension, diabetes or high blood sugar, cancer or a malignant tumor, chronic lung disease such as chronic  bronchitis or emphysema, heart diseases, stroke, emotional, nervous, or psychiatric  problems, arthritis, dyslipidemia, liver disease, kidney disease, stomach or other digestive disease, asthma. | Total number of self-reported chronic diseases that had been diagnosed at the baseline. |
| Family wealth | It represented the total value of non-housing financial wealth that was reported by respondent and his/her spouse at the baseline. It was calculated by summing cash and saving deposits, stocks and mutual funds, government bonds and all other savings, minus the value of debt. | Total value of non-housing financial wealth at the baseline |
| Later-life Health | It measured whether the respondents have particular diseases and health risks that are closely related to dementia at the baseline, including hypertension, heart diseases, diabetes, and dyslipidemia at the baseline. A set of dummy variables was constructed to denote the diseases. | Health risks for dementia and cognitive impairment. |
| Health behaviors (i.e., Smoking, Drinking) | The respondent was asked to report their current smoking habit (i.e., still smoking) and drinking (i.e., has had an alcoholic beverage in the last 12 months). We used dummy variables to denote whether the respondent was still smoking at the baseline (0/1), and has had drinking behavior. | Smoking, and drinking habits, as measures of the respondent’s health risk behaviors. |
| Social Engagement | In each of the three CHARLS core surveys, the respondent was asked to report whether they participated in the following social activities in the past month: “Interacted with friend”, “Played Ma-jong, chess, cards, or went to a community club”, “Went to a sporting event, participated in a social group, or participated in some other sort of club”, “Took part in a community-related organization”, “Took part in voluntary or charity work”, “Attended an educational or training course”. A set of dummy variables was constructed to denote whether the respondent participated in any of these social activities in the three waves (0/1). | Social activities, as a measure of social engagement. |

*Notes*: The social engagement were constructed based on the measures collected from wave 1 to wave 3 to capture the respondents’ long-term patterns of social activities. Other mediators or covariates were constructed based on the baseline CHARLS survey (i.e., wave 1), which was fielded in 2011/2012.

**Appendix D. Cognitive Tests in CHARLS**

There were five cognitive tests measured in the CHARLS baseline and two follow-up surveys: immediate word recall, delayed word recall, serial 7s, date naming, and picture drawing. Among them, immediate and delayed word recall tests, were used to assess individuals’ short-term and long-term memory.

- **Immediate word recall.** The respondent was first asked to recall as many as words as he/she could, immediately after the interviewer read out a list of 10 words (i.e., immediate word recall, range 0-10). The interviewer recorded the number of words that the respondent remembered correctly, and the words did not have to be in the same order as the ones he/she heard
- **Delayed word recall.** Approximately 5 minutes after the respondent recall the words, he/she was asked to repeat the list of words again (i.e., delayed word recall, range: 0-10) without any hints from the interviewer. Similarly, the interviewer recorded the number of words that the respondent remembered correctly this time (and the words did not have to be in the same order as the ones)

The other three tests, serial 7’s test, date naming, and picture drawing were designed to assess the respondents’ ability to perform mathematical tasks, orientation, and mental intactness.

- **Serial 7’s.** The test asked the respondent to subtract 7 from 100 for 5 trials; and the interviewer recorded the number of correct answers (range 0-5).
- **Date naming.** The test tested whether the respondent was able to correctly report today’s date, including the day of month, month, year, as well as the day of the week (range 0-4).
- **Picture drawing.** The test assessed whether the respondent was able to redraw a picture (i.e., a picture of two pentagons overlapped) shown by the interviewer (0/1) (Ofstedal et al., 2005; Zhao et al., 2020).

These cognitive tests were conducted by interviewers who were trained with a standard and stringent protocol. Though the interviewers were mostly college or graduate students, the cognitive tests were well-designed, straightforward, and easy to conduct. In addition, the tests were largely objective evaluations, while requiring limited expertise in medical or cognitive science. Therefore, interviewers with standard training would be able to conduct the tests through face-to-face interviews (Zhao et al., 2020, 2014).

**References**

Ofstedal, M.B., Fisher, G.G., Herzog, A.R., Wallace, R., Weir, D., Langa, K., 2005. HRS/AHEAD documentation report: Documentation of cognitive functioning measures in the Health and Retirement Study. Mich. Surv. Res. Cent. Univ. Mich.

Zhao, Y., Crimmins, E.M., Hu, P., Shen, Y., Smith, J.P., Strauss, J., Wang, Y., Zhang, Y., 2016. Prevalence, diagnosis, and management of diabetes mellitus among older Chinese: results from the China Health and Retirement Longitudinal Study. Int. J. Public Health 61, 347–356.

Zhao, Y., Hu, Y., Smith, J.P., Strauss, J., Yang, G., 2014. Cohort Profile: The China Health and Retirement Longitudinal Study (CHARLS). Int. J. Epidemiol. 43, 61–68. https://doi.org/10.1093/ije/dys203

Zhao, Y., Strauss, J., Chen, X., Wang, Y., Gong, J., Meng, Q., Wang, G., Wang, H., 2020. China Health and Retirement Longitudinal Study Wave 4 User’s Guide. Natl. Sch. Dev. Peking Univ.
